# Prognostic potential of CUL3 ligase with differential roles in luminal A and basal type breast cancer tumors

**DOI:** 10.1038/s41598-024-65692-z

**Published:** 2024-06-28

**Authors:** Vasiliki Pantazi, Vanda Miklós, Paul Smith, Orsolya Oláh-Németh, Gabriella Pankotai-Bodó, Divya Teja Dondapati, Ferhan Ayaydin, Vincenzo D’Angiolella, Tibor Pankotai

**Affiliations:** 1 Genome Integrity and DNA Repair Core Group, Hungarian Centre of Excellence for Molecular Medicine (HCEMM), Szeged, Hungary; 2 Department of Pathology, Albert Szent-Györgyi Medical School, University of Szeged, Szeged, Hungary; 3 Competence Centre of the Life Sciences Cluster of the Centre of Excellence for Interdisciplinary Research, Development and Innovation, University of Szeged, Szeged, Hungary; 4 The Institute of Genetics and Cancer, University of Edinburgh, Edinburgh, UK; 5 Hungarian Centre of Excellence for Molecular Medicine (HCEMM), Functional Cell Biology and Immunology Advanced Core Facility, University of Szeged, Szeged, Hungary

**Keywords:** Breast cancer, Apoptosis, Prognostic markers

## Abstract

Breast cancer is a prevalent and significant cause of mortality in women, and manifests as six molecular subtypes. Its further histologic classification into non-invasive ductal or lobular carcinoma (DCIS) and invasive carcinoma (ILC or IDC) underscores its heterogeneity. The ubiquitin–proteasome system plays a crucial role in breast cancer, with inhibitors targeting the 26S proteasome showing promise in clinical treatment. The Cullin-RING ubiquitin ligases, including CUL3, have direct links to breast cancer. This study focuses on CUL3 as a potential biomarker, leveraging high-throughput sequencing, gene expression profiling, experimental and data analysis tools. Through comprehensive analysis using databases like GEPIA2 and UALCAN, as well as TCGA datasets, CUL3's expression and its association with prognostic values were assessed. Additionally, the impact of CUL3 overexpression was explored in MCF-7 and MDA-MB-231 breast cancer cell lines, revealing distinct differences in molecular and phenotypic characteristics. We further profiled its expression and localization in breast cancer tissues identifying prominent differences between luminal A and TNBC tumors. Conclusively, CUL3 was found to be associated with cell cycle progression, and DNA damage response, exhibiting diverse roles depending on the tumor's molecular type. It exhibits a tendency to act as an oncogene in triple-negative tumors and as a tumor suppressor in luminal A types, suggesting a potential significance in breast cancer progression and therapeutic directions.

## Introduction

Breast cancer (BRCA) stands as one of the most prevalent cancer types, ranking as the second leading cause of death among women. According to the American Cancer Society's projections for 2023, approximately 297,790 new cases of invasive breast cancer and 55,000 cases of ductal carcinoma are expected to be diagnosed in women^1^. In recent years, incidence rates have shown a 0.5% annual increase, and on average, a woman in the United States faces a 13% lifetime risk of developing breast cancer. This means that 1 out of 8 women will be diagnosed with breast cancer at some point in their lives^1^.

Breast cancer exhibits a diverse nature with six distinct molecular subtypes: luminal A [characterized by progesterone receptors (PR+), estrogen receptor (ER+), Human Epidermal Growth Factor Receptor 2 (HER2)−, and Ki67−], luminal B (ER+, HER2±, and Ki67+), HER2-positive, basal-like subtype (ER−, PR−, and HER2−, also known as Triple Negative Breast Cancer or TNBC), normal breast-like, and claudin-low type^2,3^. Alternatively, based on histologic type, breast cancer is classified as ductal or lobular carcinoma in situ (DCIS), a non-invasive or pre-invasive carcinoma, and invasive carcinoma (ILC or IDC), which involves spreading into the surrounding breast tissue^2^. While ductal carcinoma in situ (DCIS) represents an intermediate phase of breast cancer and is not immediately life-threatening, it does pose a risk of progressing to invasive breast cancer (IBC)^4^. DCIS is estimated to constitute around 20% of diagnosed breast cancers in the United States, and it is believed that 14–50% of DCIS tumors may progress to IBC if left untreated. In contrast, IDC accounts for 70–80% of all breast cancer types^5^. Therefore, the identification of biomarkers is crucial for diagnosing breast cancer, distinguishing between its various types and stages, and assessing the likelihood of progression to a more invasive and aggressive phenotype.

The treatment of breast cancer is personalized based on its type and stage, involving local interventions like radiation and mastectomy, along with systemic therapies and medications^6,7^. Both short- and long-term side effects, such as hair loss, weakness, fatigue, weight gain, and infection risks, are associated with these treatments. Mastectomy also has a significant psychological impact on mental health. While mammography is a common diagnostic method, alternative techniques like magnetic resonance imaging (MRI), positron-emission tomography (PET), computed tomography (CT), and single-photon emission computed tomography (SPECT) are also used. However, mammography has limitations, such as low sensitivity in younger women with dense breast tissue and a 1–10% misdiagnosis rate^8^. Therefore, it is crucial to implement comprehensive and accurate diagnostic methods for the early detection and treatment of breast cancer. Biomarkers play a vital role in diagnosing, prognosticating, and predicting breast cancer, ensuring timely identification and effective disease management throughout the treatment process. These biomarkers can encompass various macromolecules, including nucleic acids (DNA/RNA), small extracellular vesicles as well as proteins^9,10^.

Breast cancer has long been related to the selective proteolytic ubiquitin–proteasome system (UPS)^11–13^. Ubiquitin-mediated protein degradation plays a critical role in regulating many diverse cellular processes and participates in both physiological and pathological processes of breast cancer. This regulatory system uses a series of enzymes to transfer ubiquitin molecules to their substrates to target them for destruction through proteasome machinery^14–16^. Combining inhibitors that target the 26S proteasome with other drugs has demonstrated promising therapeutic effects in the clinical management of breast cancer with novel prognostic biomarkers associated with the UPS^11,12^.

The Cullin-RING ubiquitin ligases (CRLs) are the largest class of E3 ligases, and they all contain a cullin protein as a scaffold, a RING finger protein and a substrate recognition subunit called adapter^17^. Certain members of this group, Cullin-3 (CUL3), Cullin-4 (CUL4), or Cullin-5 (CUL5) have been directly linked to breast cancer yet there are studies that exhibit conflicting findings regarding the role of CUL3^18–22^. Henceforth, in this article we are focusing on CUL3 and its prognostic value in breast cancer. With the extensive application of high-throughput sequencing and gene expression profiling as well as the plethora of data available online it has become more and more convenient to explore and identify tumor molecular markers. Using these tools, we try to elucidate the importance of CUL3 in breast cancer as a possible biomarker. We comprehensively analyzed CUL3 expression and association with prognostic values of cancer patients in breast cancer via various databases such as the GEPIA2 and UALCAN, we examined its expression correlation with other genes, and we further analyzed datasets from TCGA (The Cancer Genome Atlas). We lastly examined the effect of CUL3 overexpression in two well-known breast cancer cell lines, MCF-7 and MDA-MB-231 where we found distinctive differences in molecular and phenotypic characteristics. Together with immunohistochemical analysis of breast cancer patient tissues, our findings indicate that CUL3 is linked to cell cycle progression, and DNA damage response, showcasing distinct roles and subcellular compartmentalization depending on the molecular type of the tumor. We show that it could drive cancer progression and function as an oncogene in triple negative (basal-like) tumors whereas in luminal A types shifts to a tumor suppressor role inhibiting its progression.

## Results

### CUL3 expression correlates with unique signatures in certain tumor types

**Figure 1:**
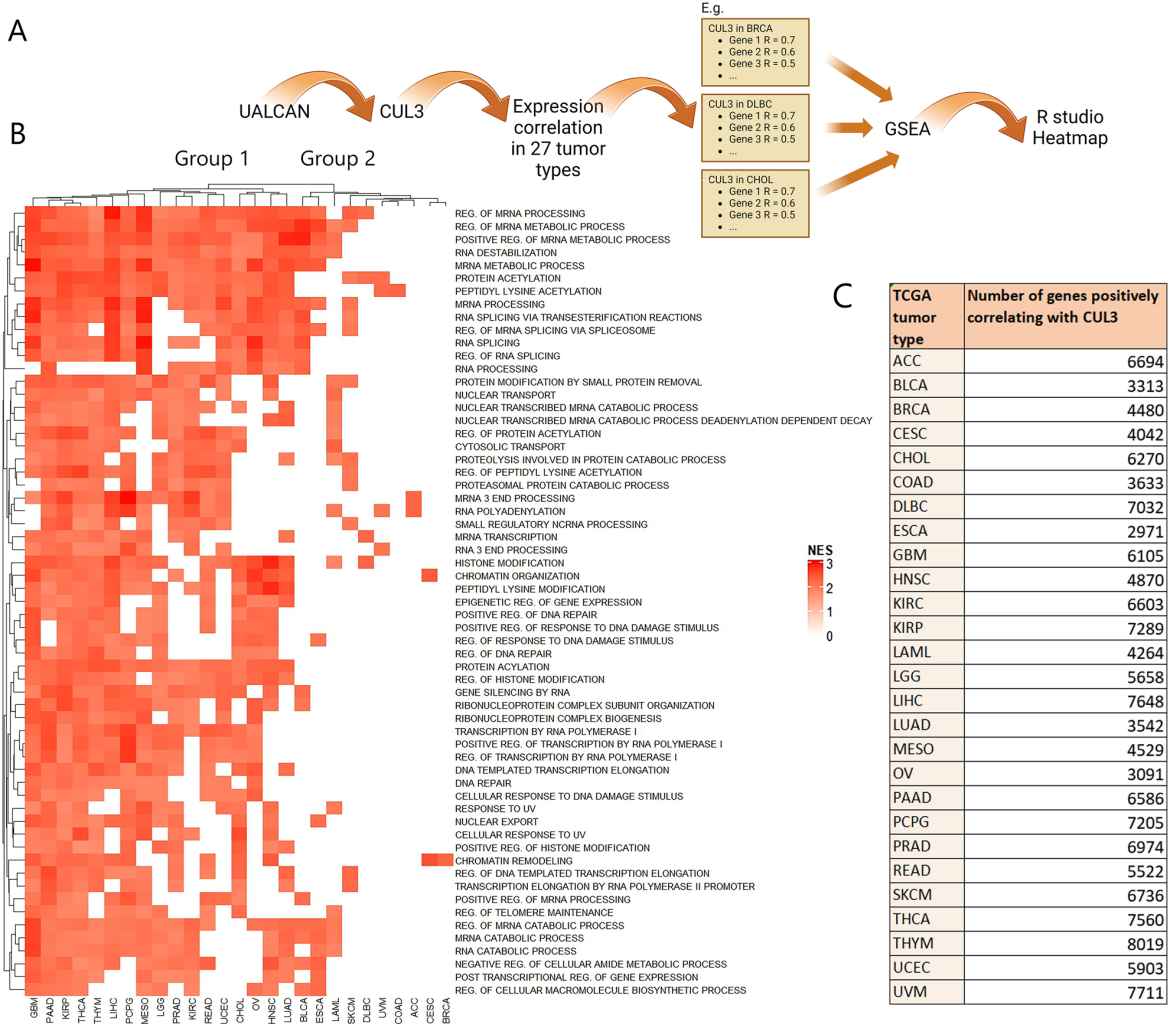
(**A**) Schematic representation of the pipeline to retrieve molecular signatures from genes positively correlating with CUL3 in 27 tumor types. CUL3 expression correlation was explored in UALCAN data portal for each of the 27 tumor types available. For every tumor type the CUL3 positively correlating genes were retrieved and all the gene lists with the respective Pearson’s correlation values per gene were submitted in GSEA and molecular signatures were enriched for every tumor. The top 60 common pathways among the 27 tumors were plotted in R studio (illustratution was created with BioRender.com) (**B**) Heatmap of GSEA pathways positively correlating with the expression levels of CUL3 in 27 tumor types. The heatmap was generated with the ComplexHeatmap (version 2.18.0; https://bioconductor.org/packages/release/bioc/html/ComplexHeatmap.html) in R studio (R version 4.3.0). Hierarchical clustering of rows and columns was based on Euclidean distance. *NES*; normalized enrichment score portraying the spread of GSEA pathways among the 27 gene lists ranked by hypergeometric score (HGS). (**C**) Number of genes per tumor type that positively correlated with CUL3 (retrieved from UALCAN) and were used for GSEA.

The cullin-RING family (CRL) is the largest family of E3 ligases in mammals comprising of more than 200 multi-subunit E3 ubiquitin ligase complexes assembled from the eight different scaffold proteins in the cullin protein family (CUL1, CUL2, CUL3, CUL4a, CUL4b, CUL5, CUL7, CUL9). CUL3 E3 ligase complex as well as the other cullin members catalyze the ubiquitylation of many substrate proteins and since ubiquitylation is an indispensable pathway regulating the metabolic programming of various diseases including cancer we wanted to examine how the expression of CUL3 correlates with molecular pathways in different tumor types. Correlation analysis of genes using RNA expression values is a valuable tool to identify interacting genes or co-expressing genes and regulatory networks. For this reason, we retrieved genes that were positively correlating (Pearson’s correlation ≥ 0.3) with the expression levels of *CUL3* from the web-portal UALCAN that provides access to publicly available RNA-seq (level 3) TCGA data from 31 cancer types. We were able to retrieve the genes that were positively correlating with *CUL3* from 27 tumor types (Fig. 1C) and using these gene lists we performed GSEA (Gene Set Enrichment Analysis) to enrich molecular signatures that these genes participate in. We then plotted in a heatmap the top 60 common pathways filtering out those with FDR q-value lower than 0.1 as shown in Fig. 1A and B. The results showed that tumor types were clustered into 2 major groups; group 1 clustered 17 tumor types which consisted of a plethora of pathways correlating with CUL3: Glioblastoma (GBM), Pancreatic adenocarcinoma (PAAD), Kidney renal papillary cell carcinoma (KIRP), Thyroid carcinoma (THCA), Thymoma (THYM), Liver hepatocellular carcinoma (LIHC), Pheochromocytoma and paraganglioma (PCPG), Mesothelioma (MESO), Brain lower grade glioma (LGG), Prostate adenocarcinoma (PRAD), Kidney renal clear cell carcinoma (KIRC), Rectum adenocarcinoma (READ), Uterine corpus endometrial carcinoma (USEC), Cholangiocarcinoma (CHOL), Ovarian serous cystadenocarcinoma (OV), Head and Neck squamous cell carcinoma (HNSC), and Lung adenocarcinoma (LUAD). Group 2 contained 10 tumor types that exhibited limited number or “unique” correlating pathways: Bladder urothelial carcinoma (BLCA), Esophageal carcinoma (ESCA), Acute myeloid leukemia (LAML), Skin cutaneous melanoma (SKCM), Lymphoid neoplasm diffuse large B-cell lymphoma (DLBC), Uveal melanoma (UVM), Colon adenocarcinoma (COAD), Adrenocortical carcinoma (ACC), Cervical squamous cell carcinoma and endocervical adenocarcinoma (CESC), or Breast cancer (BRCA). We believe that the latter ones (Group 2) highlight the unique features the CUL3 complexes adopt in case of a specified cancer type versus normal tissues and raises the question of whether CUL3 is an indispensable novel element in case of tumor types of Group 2 raising the demand for further exploration and validation. Additionally, we noted that specifically in BRCA and in cervical squamous cell carcinoma (CESC), among all the common signatures of the various tumor types CUL3 seems to correlate well only with chromatin remodeling pathways. It seems that this was the only common enriched pathway involving CUL3 among the various cancer types. On the other hand, in adenoid cystic carcinoma (ACC) we notice only mRNA processing biological pathways. We reason that Group 2 is of particular interest highlighting the unique features CUL3 might exhibit on those cancers.

### High expression of CUL3 associates with poor prognosis in BRCA

**Figure 2:**
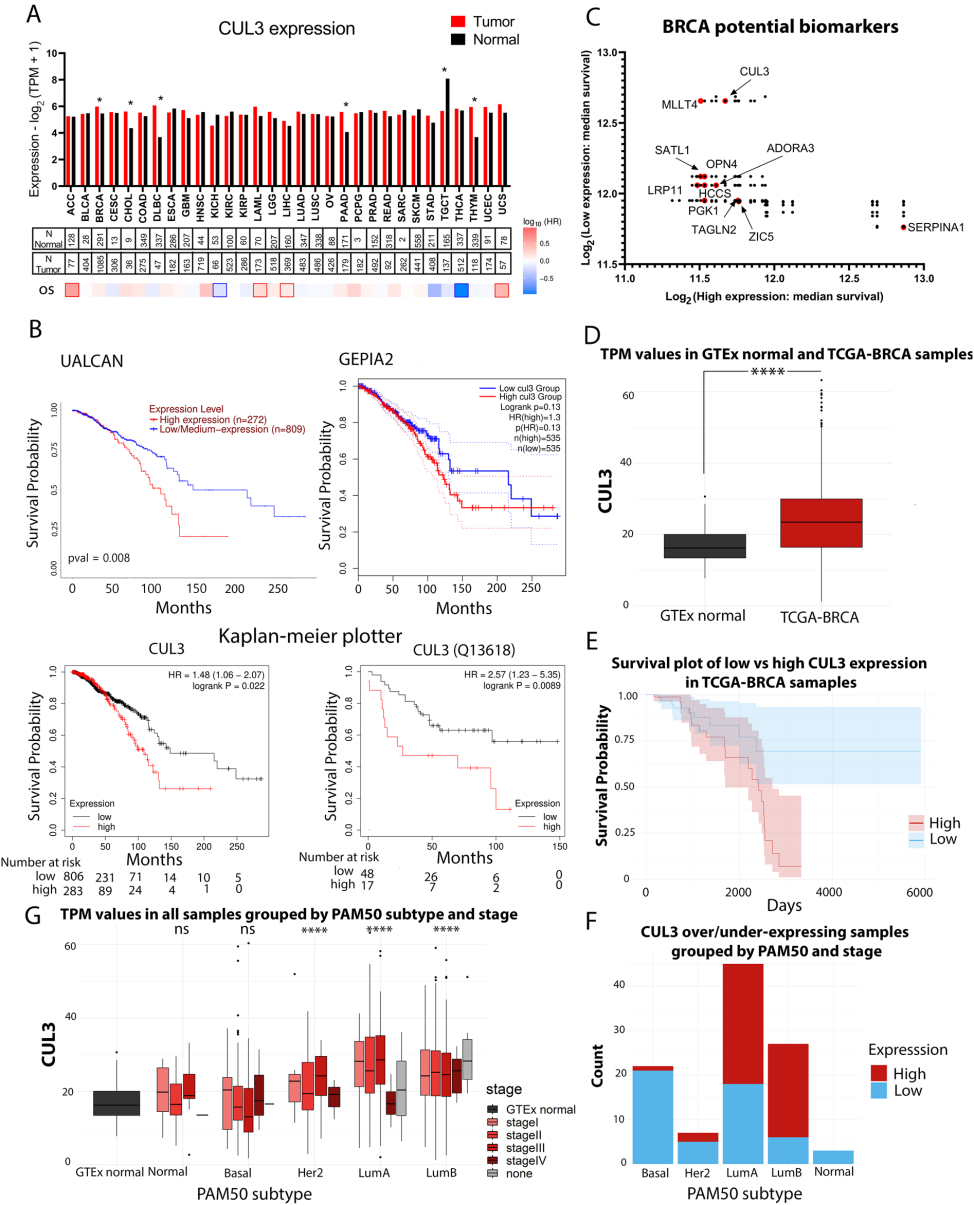
(**A**) 31 cancer types showing CUL3 differential gene expression (DGE) between normal and tumor tissues retrieved from GEPIA2. Below the number of samples quantified as well as the overall survival (OS) status with the calculated hazard ratios (HR) are shown. (**B**) Kaplan–Meier plots from three different web-portals; UALCAN, GEPIA2, Kaplan–Meier plotter depicting the survival probability of high/low CUL3 mRNA expression, whereas CUL3 (13618) plot refers to survival probability of CUL3 with high/low protein expression and (**C**) CUL3 among the breast cancer prognostic markers list retrieved from UALCAN, (**D**) TCGA analysis of CUL3 expression between breast cancer and normal tissues, (**E**) TCGA analysis of overall survival of breast cancer patients having high versus low CUL3 expression, (**F**) Breast cancer molecular subtype (PAM50 subtype) of patients involved in (**E**), and (**G**) CUL3 expression across the 5 subtypes (PAM50 subtypes) and the stages of each in breast cancer. For (**D**) and (**G**). Significance between mean TPM values were calculated by Student's t-Test in R for which *p* value was considered significant as follows: *p* < 0.1*, *p* < 0.01**, *p* < 0.001***, *p* < 0.0001****, *p* > 0.1 ns, *TPM*; transcript per million.

As stated in Fig. 1 we grouped 10 tumor types in Group 2 according to the number of biological pathways that contain genes highly associated with the expression of CUL3 bearing a limited number of enriched terms. We further examined the expression levels of CUL3 in tumor and paired normal tissues acquiring the median transcript per million values (TPM) from GEPIA2 web-portal that integrates the expression profiles of genes from RNA-seq data of the TCGA and GTEx projects (Fig. 2A) and noticed that CUL3 is significantly upregulated in the cases of Breast cancer (BRCA), Cholangiocarcinoma (CHOL), Diffuse large B cell Lymphoma (DLBC), Pancreatic adenocarcinoma (PAAD), and thymoma (THYM) whereas significantly downregulated in testicular germ cell tumor (TGCT). Using additional web-portals, we cross examined the expression levels of CUL3 across the above-mentioned tumor types (UALCAN; Supplementary Fig. S1A) and normal vs tumor tissues were compared (AnalyseR; Supplementary Fig. S1B). We did notice differences between the statistical significance in the different portals, but this might be due to either differences in data curation, bioinformatic pipelines or the incorporation of additional information from the GTEx project that provides gene expression data from healthy, cancer-free individuals providing stronger, yet somewhat different statistical power.

We further investigated the correlation between CUL3 expression and survival probability in tumor types belonging to Group 2, specifically DLBC and BRCA, that had statistically significant differential CUL3 expression among the 10 tumor types of Group 2 (Fig. 2A, see asterisks). In BRCA, elevated CUL3 expression was associated with a poorer prognosis, leading to a faster reduction in overall survival probability (Fig. 2B). This finding was confirmed across three different web portals and identified CUL3 among potential prognostic biomarkers in breast cancer, ranked by their significance in overall survival rates (Fig. 2C). Our “in-house” in silico analysis of TCGA data affirmed robustly high CUL3 expression in breast cancer compared to normal samples, correlating with a poorer prognosis (Fig. 2D,E). Notably, samples contributing to the survival plot with high CUL3 expression predominantly belonged to Luminal A, B, and Basal types, the most common molecular subtypes of BRCA. Furthermore, Luminal A and B tumors exhibited higher CUL3 expression, while Basal or Her2 types showed no significant difference in sample distribution (Fig. 2F). We then examined CUL3 expression across various molecular subtypes of breast cancer and their stages, comparing them to healthy mammary tissue. Luminal A, B, and Her2 subtypes demonstrated higher CUL3 expression levels compared to normal mammary tissue, while normal-like and basal subtypes resembled healthy tissues (Fig. 2G). Intriguingly, stage IV exhibited contrasting trends between Luminal A/Her2 and Basal types. In this metastasized tumor stage, Luminal A or Her2 types are characterized by the lowest CUL3 expression levels, while advanced triple negative BRCA tumors exhibit high CUL3 expression (Fig. 2G).

Nevertheless, when examining DLBC cancer we were not able to identify significant prognostic value in any of the web resources potentially due to the limited number of patients involved in the studies (Supplementary Fig. S1B). However, although our TCGA analysis showed somewhat significant high CUL3 expression in DLBC samples, it did not affect the survival of the patient (Supplementary Fig. S1C–E). Therefore, these results suggest that the expression levels of CUL3 might stand as a potential prognostic factor in breast cancer patients with higher expression levels correlating with lower chances of recovery.

### CUL3 participates in excessive proliferation and DNA damage response in BRCA

**Figure 3:**
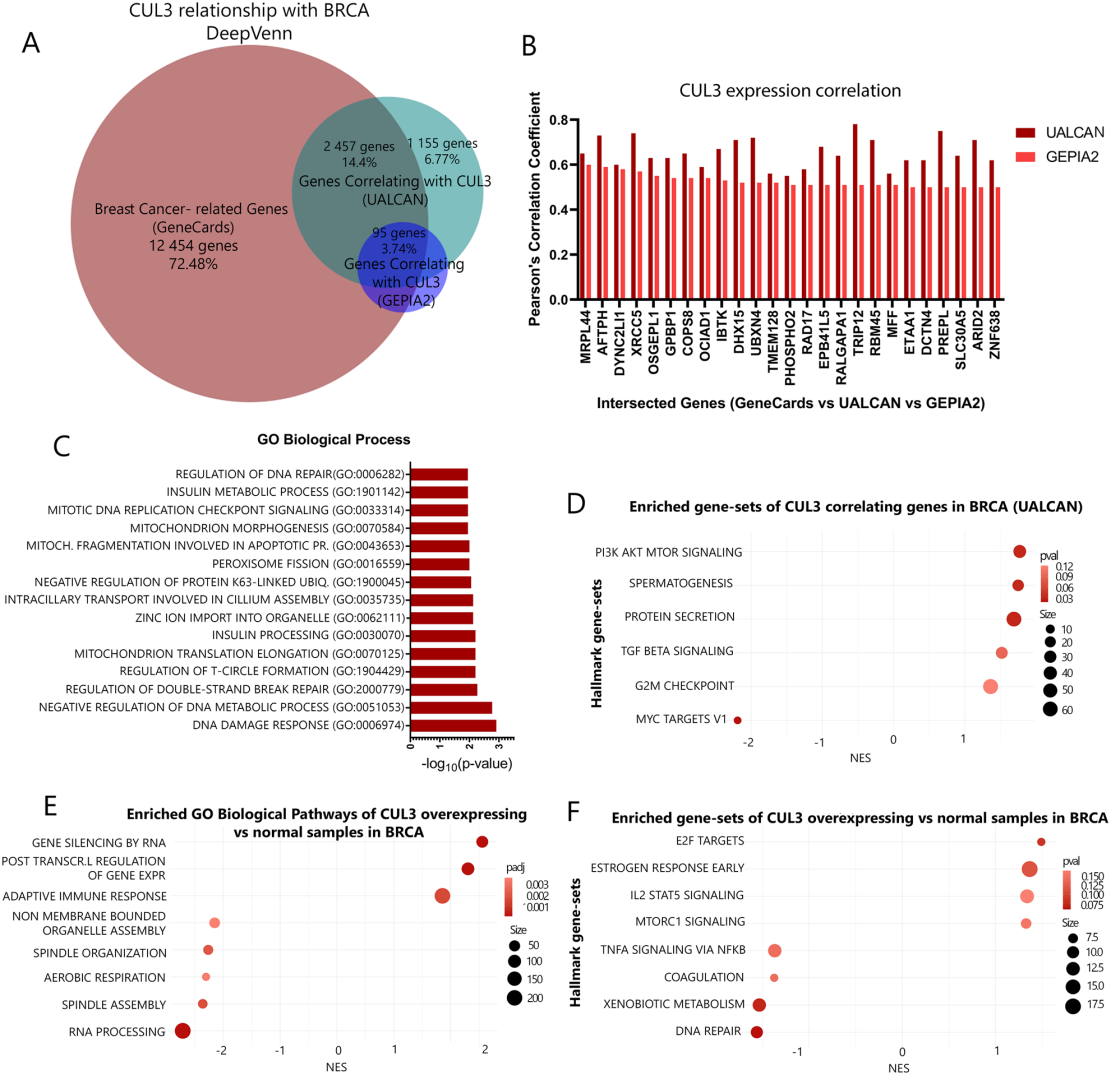
(**A**) Venn diagram showing the intersections between genes related to breast cancer (according to GeneCards database) and genes correlating expression wise with CUL3 from UALCAN and GEPIA2, (**B**) The Pearson's correlation coefficient of the intersected genes from (**A**) (GeneCards vs UALCAN vs GEPIA2) as it is calculated in UALCAN and GEPIA2, (**C**) Gene Ontology (GO) Biological pathways enrichment analysis of the top 25 highly correlated genes enriching for biological processes of (**B**), (**D**) Hallmark gene-sets (MSigDB) of positive expression correlation genes with CUL3 in breast cancer (Pearson’s correlation coefficient, R > 0.3), (**E**) Gene Ontology Biological Pathways (GO BP) of differentially expressed genes in CUL3 overexpressing samples in breast cancer versus normal tissues and (**F**) Hallmark gene-sets (MSigDB) of differentially expressed genes in CUL3 overexpressing samples in breast cancer versus normal tissues; *NES* normalized enriched score.

Next, we aimed to assess the correlation between CUL3 expression and disease-related genes, specifically those associated with breast cancer. Retrieving these genes from GeneCards, we intersected them with CUL3-correlating genes from UALCAN (4479 genes) and GEPIA2 (638 genes), to include inter-verifiable genes using a Pearson’s correlation coefficient cutoff of ≥ 0.5. The results included a set of 25 genes (Fig. 3A–B and Supplementary Fig. S2A–C).

Correlation graphs for gene pairs that showed the highest correlation (CUL3-XRCC5, CUL3-AFTPH, CUL3-MRPL44, CUL3-XRCC6, CUL3-XRCC4) were generated from three different portals; UALCAN, GEPIA2 and AnalyzeR (Supplementary Fig. S2). To validate our hypothesis, we checked correlations with unrelated control genes (GAPDH, PPIB, ACTB, B2M, TUBB2A), finding no significant correlation (R < 0.2) strengthening the notion of common gene regulation between CUL3 and the above-mentioned genes. Using these top 25 genes in EnrichR to retrieve Gene Ontology biological processes, we found significant enrichment in DNA damage response (DDR) and mitochondrial pathways (Fig. 3C). Since XRCC5 in DDR pathways had the highest correlation with CUL3, we also examined CUL3 expression correlation with XRCC4 and XRCC6 which are core members of the same network, NHEJ, but yielded weaker correlations (Supplementary Fig. S2).

Examining Hallmark gene-sets (MSigDB) for positively and negatively correlating genes with CUL3 in breast cancer (Pearson’s coefficient > 0.3 or − 0.3), we identified pathways related to cell proliferation, survival, metabolism, growth, and differentiation, including PI3K/Akt/mTOR, TGFβ, and G2/M damage checkpoint pathways. Notably, a negative correlation with MYC targets V1, associated with cancer aggressiveness and poor survival in ER+ and metastatic breast cancers, was observed (Fig. 3D).

Additionally, we took the differentially expressed genes (both up and down regulated) in overexpressed CUL3 breast cancer samples and ordered them based on log_2_ fold change values. The ordered gene list was used as the input for the Hallmark (MSigDB) gene set enrichment analysis as well as for Gene Ontology Biological Pathways (GO BP) and the result verified our expression correlation analysis revealing pathways involved in cell proliferation, survival and growth (E2F targets, Estrogen Response Early, MTORC1 and IL2 STAT5 signaling pathways) whereas pathways such as TNF-alpha signaling via NK-κΒ showed downregulation in CUL3 overexpressed breast tissues. Interestingly, DNA repair was also downregulated in CUL3 overexpression which can account for excessive mutational burden combined with the excessive proliferation and reduced immune response (Fig. 3F). From the GO BP terms we can distinguish a significantly reduced representation of genes involved in mitotic spindle assembly and organization (Fig. 3E). When considered alongside this data, we postulate that the heightened expression of CUL3, linked to unfavorable BRCA prognosis, might be connected to the disruption of double-strand break (DSB) repair mechanisms. This disruption could potentially facilitate evasion of apoptosis, fostering uncontrolled proliferation and thereby promoting tumor growth.

### CUL3 overexpression exacerbates tumor growth in TNBC BRCA but not in luminal A

**Figure 4:**
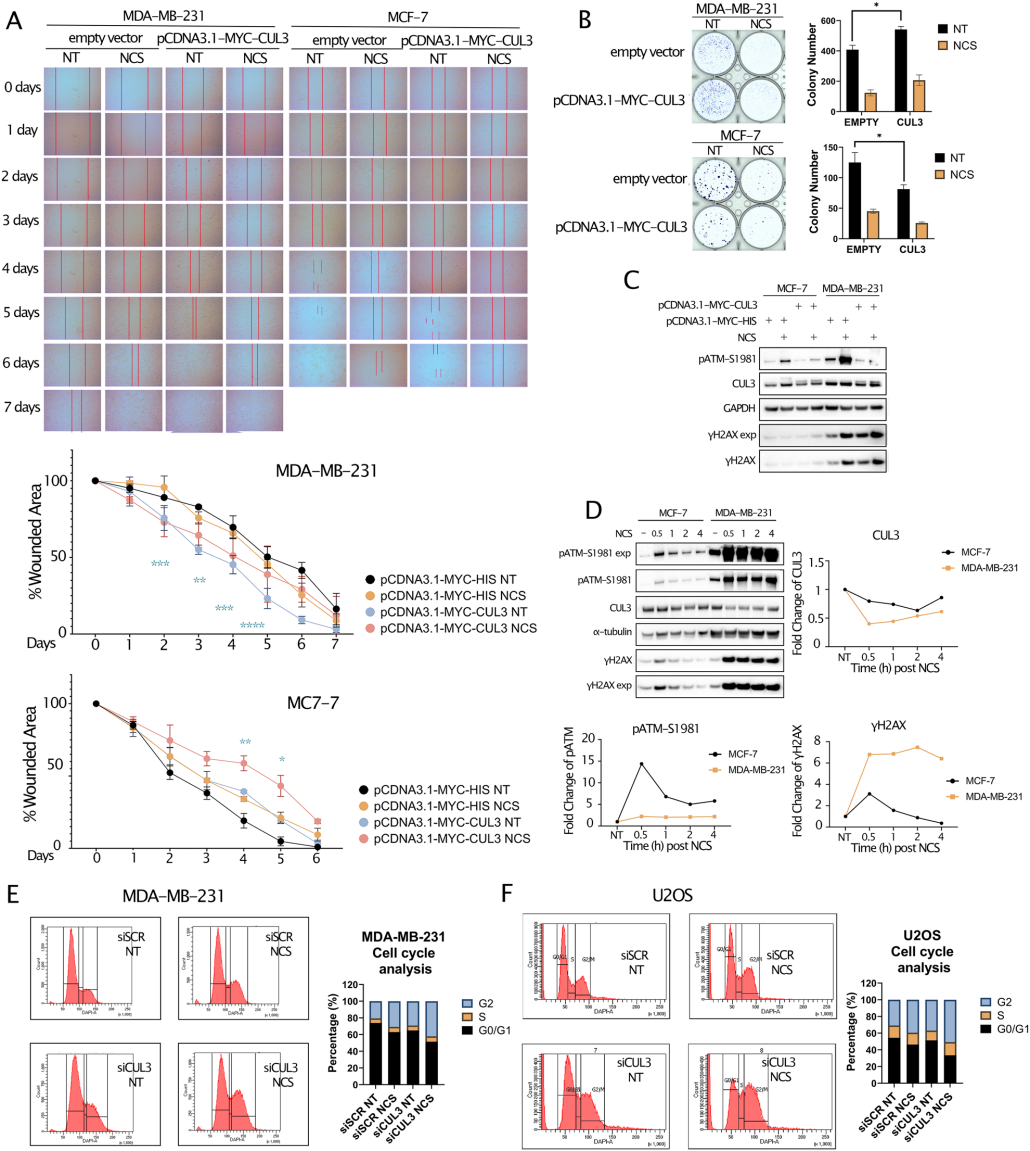
(**A**) Top Microscopic images of wound healing assay and bottom quantification of the wounded area per cell line, (**B**) colony formation assay and quantification on the right (**C**) Immunoblotting analysis of (**A**) and (**B**) in MCF-7 and MDA-MB-231 cell lines after overexpression of either MYC-HIS (empty) vector or MYC-CUL3 and treatment with NCS, (**D**) Left Immunoblotting analysis of MCF-7 and MDA-MB-231 cells after the indicated time-points of NCS treatment and right and bottom quantification of fold change of target proteins (pATM-S1981, CUL3 and γH2AX) after normalization to Ponceau staining, (**E**) Left flow cytometry images of cell cycle analysis of MDA-MB-231 after siCUL3 knock-down and 6 h NCS treatment and Right quantification, (**F**) Left Flow cytometry images of cell cycle analysis of U2OS after siCUL3 knock-down and 6 h NCS treatment and Right quantification. All quantifications were performed using GraphPad Prism version 8.4.3 (https://www.graphpad.com/) software and Tukey’s test two-way Anova was applied for statistical analysis for which *p* value was considered significant as follows: *0.0332, **0.0021, ***0.0002, ****< 0.0001.

We extended our investigation to assess the impact of CUL3 in two prominent human breast cancer cell lines, MCF-7 and MDA-MB-231, characterized by distinct molecular and phenotypic traits. MCF-7, originating from luminal A epithelial ductal carcinoma, is hormone-sensitive and less aggressive, while MDA-MB-231 is a highly aggressive triple-negative breast cancer (TNBC) cell line^23–25^. Notably, these differences in molecular characteristics prompted us to hypothesize that CUL3 overexpression might yield diverse cellular and phenotypic outcomes. According to Fig. 2G we could see a distinction between the Luminal A and Basal (TNBC) tumors in the expression of endogenous CUL3 level. For this reason, we hypothesize that the CUL3 overexpression could result in different cellular or even phenotypic differences. To unravel this and to mimic higher expression patterns, we first transfected both cell lines with pCDNA3.1-MYC-CUL3 and pCDNA3.1-MYC-HIS vector as a control and monitored for 5–6 days their migration ability after the induction of a wound as well as DNA damage using neocarzinostatin (NCS; Fig. 4A). NCS is a radiomimetic drug that can induce DNA double-strand breaks (verified by the colocalization of γH2AX and 53BP1 proteins; Supplementary Fig. S5B) and since CUL3 overexpression demonstrated reverse correlation with DNA damage response, from our previous results, we wanted to examine the synthetic effect of those. We noticed that in MDA-MB-231 cells the overexpression of CUL3 led to significant cellular migration whereas in the MCF-7 cell line the wound closure was delayed. As expected, DNA damage could attenuate the migratory ability in both cases proving its chemotherapeutic characteristics and the detrimental effects on proliferation and migration. We also measured the growth potential of breast cancer cells after overexpressing CUL3 by performing colony formation assay. The results were in line with the wound healing assay demonstrating that the MDA-MB-231 cells harbor higher proliferation and survival potential when CUL3 ligase is overexpressed. Instead, in the MCF-7 cells this overexpression delayed colony formation (Fig. 4B). Surprisingly, the combined treatment of CUL3 overexpression and NCS in MCF-7 cells immensely decreased colony formation as well as wound closure speculating a synthetic potential anti-cancer treatment role in those cell types. We further performed the same experimental setup after CUL3 KD where we indeed noticed the reverse effects in both cells lines as compared to the overexpression of CUL3 strengthening the notion of CUL3 specificity and its central role in regulation of proliferation (Supplementary Fig. S4).

Considering the substantial and remarkable differences between the two breast cancer cell lines in response to CUL3 overexpression we wanted to examine whether they respond differently in exposure to NCS treatment by checking certain DDR markers and the endogenous CUL3 protein level (Fig. 4D). These markers are γH2AX which denotes the phosphorylated histone H2AX at its serine 139 as well as the phosphorylated ATM kinase at serine 1981, both widely used in the field of DNA repair as markers of DSB induction. We found that the TNBC-derived cell line had substantially increased γH2AX protein level even after 4 h post NCS treatment compared to the MCF-7 cells that exhibited reduction in γH2AX protein level immediately after 30 min treatment. Furthermore, the γH2AX protein level was in line with the protein level of its upstream DDR regulator ATM kinase which also retained its phosphorylation and therefore its activation in MDA-MB-231 cells after 4 h NCS treatment as opposed to MCF-7. Remarkably, CUL3 was highly reduced in MDA cells in response to DNA damage with a more moderate effect observed in MCF-7 (Fig. 4D). Nonetheless, its overexpression led to reduced ATM phosphorylation but not γH2AX in both cell lines, with a more robust effect in MDA cells, speculating that higher CUL3 expression during DNA damage restrains proper DDR in both tumor types (Fig. 4C).

We further performed  FACS analysis experiments to measure the cell cycle after CUL3 knock-down in MDA-MB-231 as well in the U2OS osteosarcoma cell line which exhibited the same oncogenic phenotypic characteristics after CUL3 overexpression as in case of MDA-MB-231 cells (Supplementary Fig. S3A). We were able to detect a much higher number of cells arrested in G2/M phase after CUL3 transient knock-down and even stronger cell arrest after the synthetic effect of CUL3 silencing and DNA damage induction in both cell lines indicating an increased difficulty in repairing DNA and halting the cell cycle advancement (Fig. 4E,F and Supplementary Fig. S3E). Since NCS is a radiomimetic drug, we also monitored the proliferation ability of cells after increasing doses of ionizing radiation treatment in U2OS cells (Supplementary Fig. S3B,F). To do this and to overcome the transient effect of siRNAs, we first constructed a DOX inducible stable cell line expressing shRNA to efficiently knock-down CUL3, hereafter called U2OS-shCUL3 (Supplementary Fig. S3C,D). The proliferation ability of cells after DOX addition and IR exposure was significantly halted in the first days of the experiment and proceeded to a complete loss of viability after 7 days time-course indicating that cell proliferation and survival is CUL3 and IR-dependent exhibiting a synthetic effect presumably through maintaining correct G2/M phase checkpoint and allowing a fine-tuned mitosis (Fig. 4E,F, Supplementary Video 1). Collectively our results demonstrate distinctly opposite phenotypes between the two breast cancer cell lines granting differential roles to CUL3 in breast tumor progression and link CUL3 ligase complexes with the cell cycle damage checkpoint G2/M.

### CUL3 demonstrates unique subcellular localization patterns between TNBC and luminal A/B tumors

**Figure 5:**
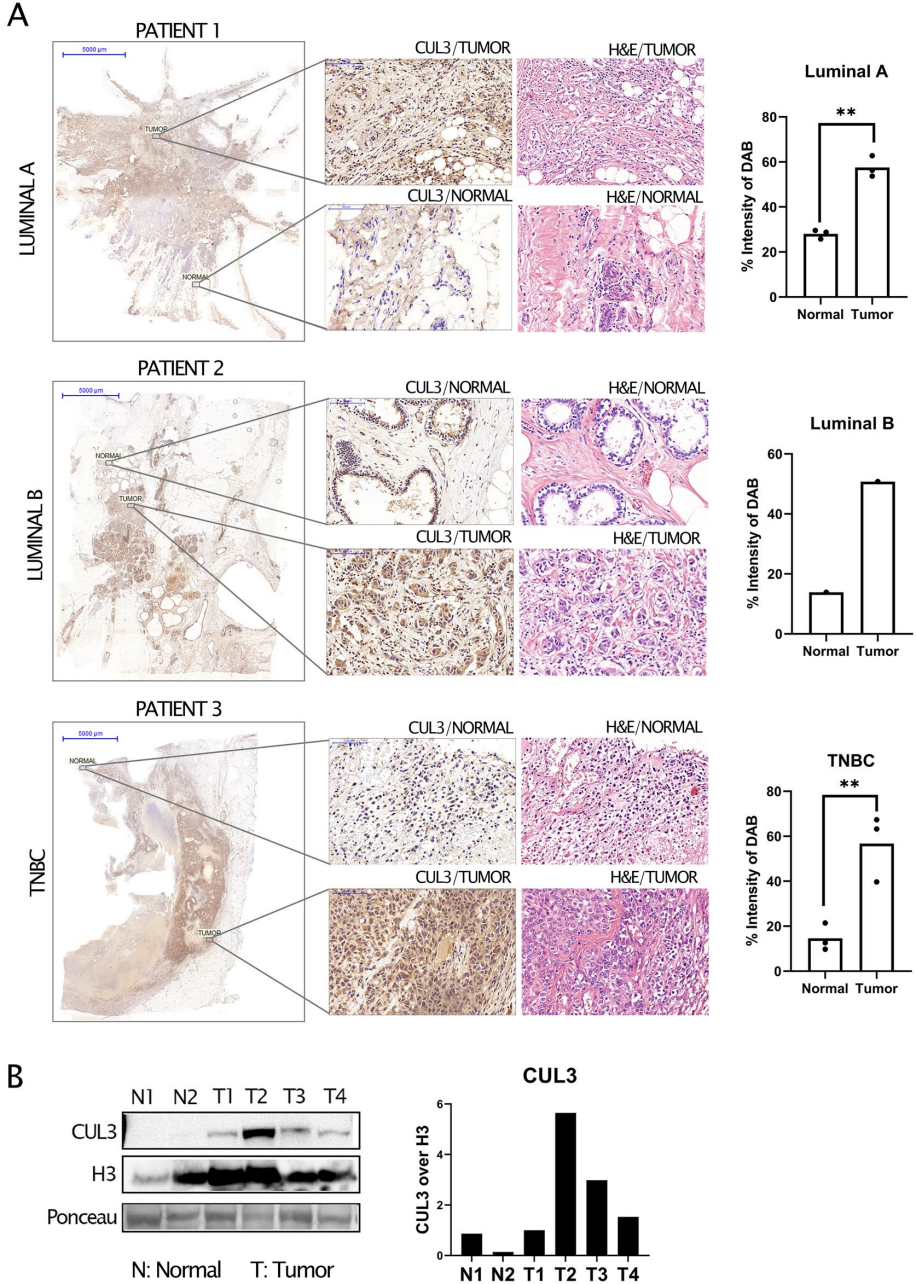
(**A**) CUL3 expression and localization in breast tissues of luminal A (top), luminal B (middle) and TNBC (bottom) after immunohistochemistry (IHC) with DAB chromogen staining (brown; staining CUL3) and Hematoxylin (blue; counter staining nuclei), parallel Hematoxylin and Eosin (H&E) staining of the same tissue and quantification of DAB intensity after color deconvolution through Image J. 3 samples were used per tumor type for the quantification (see Supplementary Fig. S5C) (**B**) Left Immunoblotting analysis of CUL3 protein level in 4 breast cancer tissues (T) and 2 healthy regions (N) and Right quantification and normalization to H3. All quantifications were performed using GraphPad Prism version 8.4.3 software (https://www.graphpad.com/) and unpaired parametric t test with Welch’s correction for SD was applied for statistical analysis for which *p* value was considered significant as follows: *0.0332, **0.0021, ***0.0002, ****< 0.0001.

Immunohistochemical analysis of CUL3 expression and localization in breast tissues of Luminal A, Luminal B and TNBC showed remarkable differences in the expression of CUL3 between the normal and tumor areas with strong CUL3 staining in the tumor ones which was also verified by immunoblotting (Fig. 5B). Therein, after tissue sampling from surgically removed breasts harboring breast cancer (see patient data in Supplementary Table S1) we detected CUL3 protein level significantly higher in the tumor region as opposed to healthy breast tissue making CUL3 ligase a potential biomarker of BRCA (Fig. 5A,B).

We could also detect pronounced discrepancies in the localization of CUL3 between the tumor types tested as well as the non-tumorous areas (determined by an experienced pathologist). Lymphocytes mainly displayed nuclear expression with a mild cytoplasmic localization; yet not all inflammatory cells were stained. In addition, epithelial cells exhibited a moderate cytoplasmic expression of CUL3. In luminal A (usually associated with lower histological grades) and luminal B tumors we detected a strong cytoplasmic CUL3 expression yet in the MCF-7 cell line immunocytochemistry staining we detected CUL3 in both nuclear and cytoplasmic regions (Fig. 5A and Supplementary Fig. S5A,C). In contrast, TNBC tumors (usually associated with higher histological grades) exhibited even stronger CUL3 staining, which was localized in perinuclear and intranuclear regions with granular staining, as well as in cytoplasmic areas verified also by our immunocytochemistry staining of MDA-MB-231 staining (Fig. 5A Supplementary Fig. S5A,C).

If a protein is found in the nucleus, it could be potentially involved in regulating gene expression, DNA repair, or cell cycle control. As in the case of TNBC, this nuclear localization could suggest a role in promoting aggressive cancer behaviors, such as rapid proliferation or resistance to apoptosis as it is also indicated in our experiments above. On the other hand, in the cytoplasm, the protein might be involved in signaling pathways, cellular metabolism, or structural functions and as we observe in Fig. 5A in luminal A breast cancer, which is typically less aggressive, cytoplasmic localization might indicate a role in maintaining normal cell function and growth regulation.

### Breast *cancer* tumors of basal and luminal A types, characterized by CUL3 overexpression, display distinct pathway enrichment and divergent patient outcomes

**Figure 6:**
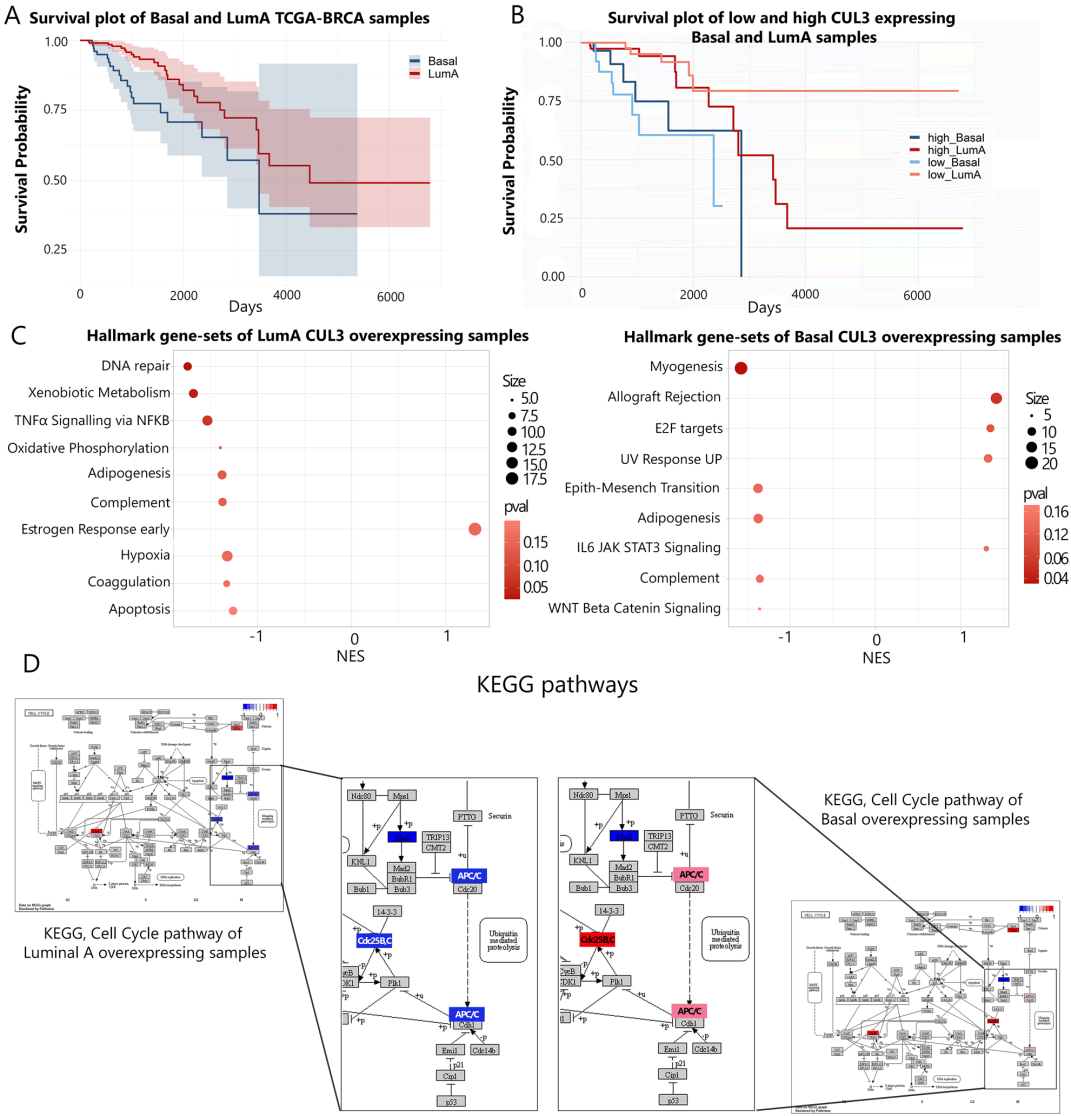
(**A**) Kaplan–meier plot of Basal and Luminal A TCGA-BRCA samples, (**B**) Kaplan-meir plot of CUL3 over- and under-expressed Basal and Luminal A TCGA-BRCA samples, (**C**) left Hallmark Gene-set enrichment of Luminal A type BRCA samples with CUL3 overexpression, right Hallmark Gene-set enrichment of Basal type BRCA samples with CUL3 overexpression and, (**D**) Differences of CUL3 overexpressed Luminal A (left) and Basal (right) type BRCA in Cell cycle after KEGG pathway analysis (hsa04110); *NES* normalized enriched score.

Building upon our earlier findings demonstrating a divergent role of the CUL3 ligase in MDA-MB-231 and MCF-7 cells, our subsequent investigation aimed to explore whether CUL3 expression correlates with distinct pathways in triple-negative breast cancer (TNBC or basal) and luminal A tumors, the origins of our studied cell lines. To accomplish this, we explored breast cancer RNA-seq datasets from TCGA, comparing the Kaplan–Meier curves of Luminal A versus Basal tumor types. As anticipated, Luminal A tumors exhibited a more favorable survival prognosis than Basal tumors despite their non-distinctive clustering after PCA plot analysis, confirming our previous observations (Fig. 6A, Supplementary Fig. S3G left). However, when we stratified these tumor types based on their CUL3 expression, we observed no significant differences within the same tumor type or differential clustering in PCA analysis (Luminal A low vs. high CUL3 expression *p* = 0.043, Basal low vs. high CUL3 expression not significant), likely due to the limited number of samples (Fig. 6B and Supplementary Fig. S3G right, Table 1). Further exploration of pathways associated with CUL3 overexpression in Basal and Luminal A tumors, utilizing the Molecular Signatures Database (MSigDB) hallmark collection, revealed distinctive signatures for each breast cancer subtype. In Basal tumors, E2F targets, allograft rejection, UV response UP, and IL6 JAK STAT3 molecular signatures were significantly positively enriched. These pathways have been linked to proliferation, breast cancer metastasis, invasiveness, aggressive cancer biology, and continuous DNA damage response. Conversely, downregulated pathways in Basal CUL3-overexpressed tumors included myogenesis, adipogenesis, epithelial-to-mesenchymal transition (EMT), complement, and WNT beta-catenin signaling, suggesting a potential reduction in stemness, differentiation, and metastatic potential (Fig. 6C right). The downregulation of EMT and WNT signaling is particularly noteworthy, as these pathways are interlinked, with WNT signaling influencing the activation of EMT transcription factors^26^. The negative impact on adipogenesis and myogenesis pathways is also intriguing, indicating potential functional limitations in TNBC patients with overexpressed CUL3, aligning with reported reductions in body fat and skeletal muscle tissue in BRCA patients^27^. In summary, elevated CUL3 expression appears to be associated with pro-cancerous characteristics, establishing it as a potential aggressive breast cancer oncogene in TNBC tumors.

In contrast, when examining the pathways associated with elevated CUL3 expression in luminal A tumors, significant distinctions emerged between the two tumor types. Only one pathway, the early estrogen response pathway, showed positive enrichment (Fig. 6C left). Notably, this pathway is indicative of a favorable response to endocrine therapy and improved survival in both primary and metastatic ER-positive breast cancer cases^28^. Despite activating genes that support proliferation and survival, this pathway was further accompanied with reduced coagulation (blood clotting), immune response (complement pathway), oxidative phosphorylation, TNFα signaling via NF-κβ, hypoxia response, and DNA repair (lower activity of DNA repair genes making tumor cells more sensitive to therapy). This alignment reinforces the hypothesis of a more positive patient prognosis, inducing tumor suppression rather than progression and aggressiveness in Luminal A BRCA tumor types. Consequently, CUL3 expression in Luminal A tumors might possibly serve as a prognostic and/or predictive biomarker for endocrine therapy, a gold standard for ER-positive/HER2-negative breast cancer, such as Luminal A.

However, these findings do not fully elucidate our previous experimental findings. Therefore, we mined into KEGG pathway analysis that revealed a significant difference in only one pathway between overexpressed CUL3 in Basal and Luminal A tumors—the cell cycle (Fig. 6D). Representative networks of the cell cycle indicated noteworthy differences, particularly in the differential regulation of key proteins such as APC, Cdc25B, and Cdc25C in the two BRCA types when CUL3 is overexpressed, suggesting an indirect link between the ligase and its substrates (Fig. 6D). Cdc25B and C, vital phosphatases at the G2/M damage checkpoint, play a crucial role in cell cycle progression. Our analysis proposes that CUL3 overexpression in Basal BRCA tumors might circumvent the G2/M damage checkpoint, potentially through degrading substrates, resulting in the accumulation and overactivation of Cdc25B, C proteins and the APC complex. This process could lead to excessive proliferation and a potential mutational burden, aligning with our experimental findings in CUL3 overexpressed MDA-MB-231 and CUL3 knock-down cell cycle assays, establishing a direct connection between the CUL3 ligase complex and cell cycle regulation. Conversely, in Luminal A BRCA tumors, these phosphatases and the APC complex are downregulated, likely inducing cell cycle arrest at the G2/M damage checkpoint. Prolonged arrest can lead to apoptosis, consistent with the observed MCF-7 phenotype upon CUL3 overexpression. Overall, despite the endogenous CUL3 expression differences in Basal and Luminal A tumors, patients with higher CUL3 expression and Basal type BRCA tumors may experience a worse prognosis possibly due to cell cycle dysregulation, leading to a more aggressive phenotype. In contrast, patients with Luminal A type and high CUL3 expression may benefit from better prognosis and potentially more effective endocrine therapy.

## Discussion

**Figure 7:**
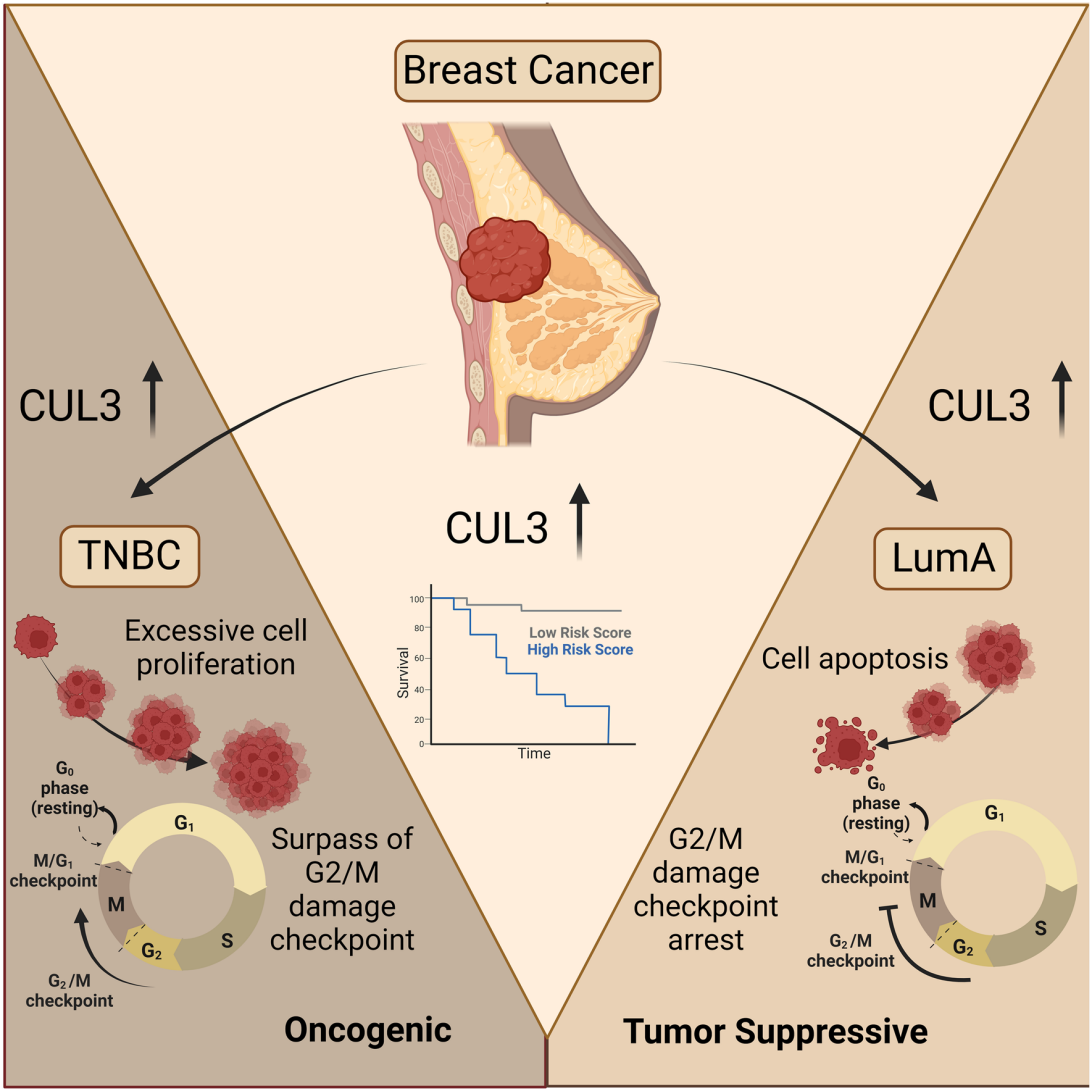
CUL3 ligase exhibits molecular function heterogeneity intratumorally. In the context of Luminal A (right panel) breast cancer, CUL3 assumes a role akin to a tumor suppressor, limiting cellular proliferation and enhancing overall survival. However, in the case of triple-negative breast cancer (left panel), CUL3 transitions into an oncogenic player, fueling cell cycle progression, fostering tumor growth, and ultimately exacerbating patient prognosis. Image was created with BioRender.com.

CUL3 ligase complexes are vital for various biological processes, participating in cell cycle regulation, stress response, transcription, and signal transduction, etc^29^. This study explores CUL3 expression across different tumors, revealing a unique prognostic potential in breast cancer. For the first time, it highlights context-dependent functions of CUL3 within specific cancer types. In Luminal A breast cancers, CUL3 acts as a tumor suppressor, reducing cell growth and enhancing survival with a primary cytoplasmic localization. Conversely, in triple-negative aggressive breast cancers, CUL3 switches to an oncogene, promoting cell cycle progression, cancer growth, exhibiting a preference in nuclear/perinuclear localization and resulting in a worse patient prognosis (Fig. 7).

CUL3 has long been related to cancer development and since most genes do not function in isolation but belong to circuits and networks that facilitate the gene’s function and subsequently catalyze the biological cellular needs, we reasoned that CUL3 could correlate expression-wise with tumor-specific biological pathways. Our expression correlation analysis heatmap could distinguish a tumor group that exhibits a very limited amount of common enriched biological terms between the tumor types, and we hypothesize that in those cancer types CUL3 demonstrates unique, distinct roles accounting for heterogeneity and diverse biological behaviors. Our investigation distinguished breast cancer as the most relevant and interesting one to study with the highest prognostic potential. In breast cancer context, CUL3 has been reported to promote tumor progression through a variety of different ways: CUL3-mediated BECLIN degradation and autophagy inhibition, CUL3-KCTD10-mediated degradation of RhoB and Rac1 activation in Her2 positive BRCA tumors, CUL3-KEAP1-NRF2 axis, CUL3-RHOBTB3- mediated regulation of COL1A1, etc^21,30–32^. Furthermore, Haagenson and colleagues generated breast cancer xenograft murine models and found that CUL3 expression correlates with breast tumor progression and stage with detectable levels even in lung metastasis further supporting our results^18^. Yet, few years later another study which was based on in-silico database (Kaplan–Meier plotter) search indicated no relation between CUL3 mRNA levels and breast cancer prognosis^22^. This might be due to the differences in the input search methods and the data curration. Herein, we chose to examine breast cancer from a pancancer point of view restricting the results only to examine the overall survival in breast cancer patients with low and high expression of CUL3 providing us with a more robust effect. On the other hand, the navigation within the breast cancer RNA-seq libraries that are provided in Kaplan–Meier plotter dataportal generate non-significant results for CUL3 expression (data not shown). In addition, there have been reports suggesting that CUL3 exhibits tumor suppressive effects prompting us to investigate its roles further to clarify those opposing statements^33^.

Our analysis indicated a correlation between CUL3 expression in breast cancer (BRCA) and genes involved in the G2/M damage checkpoint, along with enriched pro-proliferative pathways. Experimental investigations in two BRCA cell lines following CUL3 overexpression and DNA damage exposure revealed distinct molecular and phenotypic characteristics. Notably, triple-negative MDA-MB-231 cells exhibited a significant decrease in CUL3 expression in response to genotoxic stress, while the effect was moderate in MCF-7 cells. Intriguingly, CUL3 overexpression delayed the harmful consequences of DNA damage in MDA-MB-231 cells but intensified the effect in MCF-7 cells. To the best of our knowledge, there is no relevant study exposing differences between the subtypes of BRCA relevant to CUL3 ligase and therefore we screened and compared Luminal A and Basal type tumors from TCGA.

We found significant differences in gene expression in CUL3 over-expressing Luminal A and TNBC tumors with the biggest emphasis and observed differences in the cell cycle which dysregulation we were able to show experimentally as well. Our transient knock-down impaired the cell cycle by arresting cells in G2/M damage checkpoint which together with DNA damage exacerbated the effect. Moreover, KEGG analysis distinguished differences between overexpressed CUL3 LumA and Basal only in the cell cycle strengthening the notion of a direct connection of CUL3 with the cell cycle. Previous experimental evidence has shown that CUL3 is an important regulator of the cell cycle progression maintaining proper mitosis^34^. CUL3, together with its substrate receptor KLHL22, binds and ubiquitinates PLK1 (Polo-like kinase 1), leading to its dissociation from kinetochores and allowing SAC (Spindle assembly checkpoint) to be silenced and chromosomes to segregate^35^. CUL3 also controls normal mitotic progression through other mechanisms involving the substrate receptor KLHDC5 (KLHL42) or Cyclin E^34,36^. It is conceivable that one of these pathways or a yet to be uncovered one can be responsible for the distinctive differences of the two breast cancer subtypes.

Two substrate adaptors, KEAP1 and SPOP are the most representative cancer-related CUL3 adaptors, and both elicit dual and context-dependent roles in cancer. Under stress NRF2 evades its ubiquitylation from CUL3-KEAP1 and as a transcription factor initiates the expression of genes that control antioxidant responses, detoxification of xenobiotics and drugs, etc^37,38^. This phenomenon could explain the downregulation of the xenobiotic metabolism pathway detected in the Luminal A pathway enrichment of CUL3 overexpressed tumor samples (Figs. 3A and 6C). Nevertheless, many studies emphasize that when a tumor is formed despite the protective anti-cancer role of the CUL3-KEAP1-NRF2 axis, tumor cells can exploit this pathway and get “addictive” to gain growth and survival advantage and deal with the adverse conditions^39^. This is of particular interest because it might account for the adverse effect of CUL3 observed in our study in TNBC tumors granting these tumors a growth advantage. Taking these into account, we acknowledge the abundance of information on the roles and signaling pathways involving CUL3 and its adaptors in BRCA. However, to the best of our knowledge, no study has explored the intra- and inter- tumor differences it may manifest.

Our study is not free from limitations. Although we used multiple tools and data portals, we acknowledge that we utilized publicly available cohorts from previous studies, and this is a retrospective study with only limited amount of experimental evidence focusing specifically on cell lines and human tissues and not mouse in vivo models. Moreover, our investigation primarily relied on transcription profiles, neglecting the proteome and the functional distinctions between the comparisons. This oversight could potentially impact our findings, given that CUL3, as a ligase, plays a role in protein recycling, thereby influencing biological pathways. However, we believe that our study can provide insights into the complex and context-dependent roles of CUL3 in the different cancer types, highlighting its diverse functions in tumorigenesis and emphasizes the importance of considering inter- and intra- molecular cancer variations in gene expression.

## Methods

### Cell culture

MCF-7, MDA-MB-231, HEK293T were cultured in high glucose DMEM (Lonza, Basel, Switzerland) whereas U2OS were cultured in low glucose DMEM supplemented with 10% fetal bovine serum (FBS; Lonza, Basel, Switzerland), 1% antibiotic–antimycotic solution (Sigma-Aldrich, St. Louis, MO, USA), and 4 mM l-Glutamine (Sigma-Aldrich, St. Louis, MO, USA), and maintained at 37 °C in a humidified atmosphere with 5% CO2. MDA-MB-231 was a gift from Professor Dr. Francesca Buffa, MCF-7 and HEK293T were purchased from ATCC.

### shCUL3 stable cell line generation

The sequence for short hairpin RNA (shRNA) targeted against *CUL3* was 5′-GATTTGTCAAGGCAGTGCA-3′ (CatNo: V3SH11252-229569220; Dharmacon™ Trans-Lentiviral packaging kit, Cambridge, UK) to generate a doxycycline (DOX)-inducible knock-down cell line based on the tet-on system. 2 μg of shCUL3 lentiviral plasmid were transfected into HEK293T cells at 80% confluency in 6-well plate with 4.3 μl trans-lentiviral packaging mix (pTLA1-PAK, pTLA1-ENZ, pTLA1-ENV, pTLA1-TOFF and pTLA1-TAT/REV), 15 ul CaCl_2_ and 150 μl HBSS according to the manufacturer’s instructions to generate viral particles. The medium was refreshed 16 h after transfection with reduced serum (5%) medium, and the cell supernatant was collected after 48 h. The cleared supernatant was filtered through a 0.22-μm filter and U2OS cells were infected with lentiviral supernatant. After infection, the cells were selected using puromycin (2 μg/ml) for 10 days.

### Colony formation assay

10^6^ cells from both MCF-7 and MDA-MB-231 cell lines were seeded in 100 mm dishes and 24 h later at approximately 80% confluency cells were transfected with 10 μg of plasmid DNA of either pCDNA3.1-MYC-HIS (referred as empty vector; Addgene, Waretown, MA, USA) or pCDNA3.1-MYC-CUL3 (Addgene, Waretown, MA, USA) using JetPEI or Lipofectamine3000 transfection reagents (Invitrogen, Carlsbad, CA, USA) as it is described below or with siRNA transfection using 25 nM CUL3 siRNA SMARTpool (L-010224-00-0005; Dharmacon™ ON-Targetplus, Cambridge, UK) according to the manufacturers’ instructions. 48 h after transfection, 3000 cells were seeded in 6-well plates and were incubated for 14 days to form visible colonies of at least 50 cells. 24 h after seeding 100 ng/ml of neocarzinostatin (NCS; Sigma-Aldrich, St. Louis, MO, USA) was applied to the indicated wells. Cells were washed two times with 1 × PBS and fixed for 20 min using 4% formaldehyde with constant gentle agitation. After washing with 1 × PBS cells were stained with 0.05% crystal violet for 1 h and observed under light microscope. Images were taken using the Licor Odyssey M imaging system. Colonies were counted by ImageJ and GraphPad Prism version 8.4.3. (https://www.graphpad.com/) was used for quantification. Two-way Anova was applied for statistical analysis. The experiment was performed in three biological replicates.

### Wound healing assay

10^6^ cells from both MCF-7 and MDA-MB-231 cell lines were seeded in 100 mm dishes and 48 h later at approximately 80% confluency cells were transfected with 10 μg of plasmid DNA of either pCDNA3.1-MYC-HIS (empty vector) or pCDNA3.1-MYC-CUL3 using JetPEI or Lipofectamine3000 transfection reagents as it is described below or with siRNA transfection using 25 nM CUL3 siRNA SMARTpool (L-010224–00-0005; Dharmacon™ ON-Targetplus, Cambridge, UK) according to the manufacturers’ instructions. 48 h after transfection 1 × 10^5^ cells were seeded in 24-well and were let to recover for 24–48 h. A scratch was made using a pipette tip to create an incision-like gap. Immediately after the scratch 100 ng/ml of NCS was applied to the indicated wells. Images were taken using Axiocam ERc 5 s under ZEISS Primovert inverted microscope immediately after the created wounded area as well as after every 24 h for 7 days. Cell migration was quantified using ImageJ and expressed as the average percentage of closure of the scratched area using GraphPad Prism version 8.4.3. (https://www.graphpad.com/) Two-way Anova was applied for statistical analysis. The experiment was performed in three biological replicates.

### Proliferation assay

2 × 10^5^ U2OS-shCUL3 cells were seeded in 6-well plates in three technical replicates and 8 h after cell attachment, cells were treated with 1 μg/ml DOX (Sigma-Aldrich, St. Louis, MO, USA). 24 h after DOX addition, cells were exposed to various greys of a closed source gamma irradiator and were let to recover for 7 days. DOX was additionally added to the cells 3 days later to ensure CUL3 knock down. The IncuCyte S3 Live-Cell Analysis System (Sartorius, Michigan, MI, USA) was used for kinetic monitoring of cell growth and proliferation. The experiment was performed in three biological replicates and two-way Anova was used for statistical analysis.

### Plasmid transfection

For MCF-7 cells 10 μg of plasmid DNA (pCDNA3.1-MYC-HIS, pCDNA3.1-MYC-CUL3) were mixed with 500 μl 150 mM NaCl to create complex No1 and 16 μl JetPEI transfection reagent (PolyPlus, Sartorius, Michigan, MI, USA) were mixed with 500 μl 150 mM NaCl to create complex No2. Complexes No1 and No2 were incubated separately for 5 min and were later mixed and incubated for 20 min at RT before treating the cells with 1000 μl complexes.

For MDA-MB-231 cells the media was refreshed to reduced-serum OptiMEM (Sigma-Aldrich, St. Louis, MO, USA) before the transfection. 10 μg of plasmid DNA (pCDNA3.1-MYC-HIS, pCDNA3.1-MYC-CUL3) were mixed with 20 μl of reagent P3000 and 1000 μl reduced serum OptiMEM media to create complex No1 and 80 μl of Lipofectamine3000 were mixed with 1000 μl of reduced serum OptiMEM media to create complex No2. Complexes No1 and No2 were incubated separately for 5 min and were later mixed and incubated for 20 min at RT before treating the cells. 6 h after the transfection, the media was changed into high glucose DMEM containing 10% FBS.

### Tissue collection

10 sections of 20 μm thickness were excised from the tumor as well as from a healthy region of freshly surgically removed breasts bearing tumors and fast frozen in liquid nitrogen. For protein extraction tissues were lysed in RIPA buffer [50 mM Tris–HCL, 150 mM NaCl, 1% TritonX, 1 mM EDTA, 0.5% Na-DOC and 2% SDS, supplemented with 1 × PIC (Roche), 20 μM PR-619 DUBi (Deubiquitylase Inhibitor; Calbiochem, San Diego, CA, USA), and 1 × PhosSTOP (Roche, Basel, Switzerland)] followed by sonication of 10 cycles of 30 s ON/30 s OFF using Bioruptor Pico sonication device (Diagenode) and immunoblotting as described below. Tissues T1 and T2 derived from DCIS, tissue T3 from Mucinous breast carcinoma while tissue T4 from ILC. The study was conducted in accordance with the Declaration of Helsinki and approved by the Institutional Review Board (or Ethics Committee) of Science and Research Ethics of the Medical Research Council (protocol code: IV/5376-2/2020/EKU and date of approval: 30 June 2020). Informed consent was obtained from all subjects of this study.

### Immunocytochemistry

MDA-MB-231 and MCF-7 cells were washed with PBS and fixed with 4% formaldehyde (Sigma-Aldrich, St. Louis, MO, USA) for 20 min. After PBS washing, cells were permeabilized for 20 min in PBS containing 0.3% Triton-X-100. Non-specific staining was blocked with 5% BSA in PBST [0.1% Tween 20 in PBS] for 50 min. Cells were incubated with the following primary antibodies O/N at 4 °C: anti-CUL3 1:100 (ab194584; Abcam, Cambridge, UK), anti-γH2AX 1:100 (ab26350; Abcam, Cambridge, UK), anti-RNAPII CTD4H8 1:200 (sc-47701; Santa Cruz Biotechnology, Dallas, Texas, USA), and anti-53BP1 1:200 (ab36823; Abcam, Cambridge, UK). After washing, the following secondary antibodies were used: GAR Alexa 555 (A21429; Thermo Fisher Scientific, MA, USA) in 1:500 and GAM Alexa 488 (A11029; Thermo Fisher Scientific, MA, USA) in 1:500. Coverslips were mounted on glass slides using DAPI containing ProLong Gold Antifade reagent (Invitrogen™, CA, USA). Samples were visualised with Leica Stellaris laser scanning super-resolution confocal microscope. The same exposition time was used to capture each image.

#### Immunohistochemistry by DAB staining of paraffinized breast cancer tissues

Paraffinized breast tissues, originating from the histopathological tissue bank of the Department of Pathology (University of Szeged, Hungary), were kindly provided by Dr. Orsolya Oláh-Németh; all experiments were performed and procedures were approved in accordance with the Declaration of Helsinki and approved by the Institutional Review Board (or Ethics Committee) of Science and Research Ethics of the Medical Research Council (protocol code: IV/5376-2/2020/EKU and date of approval: 30 June 2020). Informed consent was obtained from all subjects of this study. Immunohistochemistry (IHC) was conducted using Leica Biosystems BOND-MAX Fully Automated IHC Staining System. BOND dewax solution (AR9222; Leica Microsystem, Wetzlar, Germany) was used 2× for 30 s at 72 °C followed by 3 × 100% Ethanol wash. BOND wash solution (AR9590; Leica Microsystem, Wetzlar, Germany) was used 2× for 5 min and then BOND epitope retrieval solution 2 (AR9640; Leica Microsystem, Wetzlar, Germany) was applied for 20 min at 100 °C with a change for 12 min. BOND-PRIME Polymer DAB Detection System kit (DS9284, Leica Microsystem, Wetzlar, Germany) was used for the following steps using primary antibody against CUL3 in 1:200 (ab194584; Abcam, Cambridge, UK). Slides were scanned using 3DHISTECH Pannoramic MIDI II scanner and figures were generated in Panoramic Viewer Software version 1.15.4. (https://www.3dhistech.com/downloads/pannoramic-viewer-1-15-4-for-windows/) The analysis was performed in Image J using color deconvolution plugin.

### Western blot

6 × 10^5^ cells were seeded in 60 mm dishes and either transfected with plasmids or treated with 100 ng/ml NCS for the indicated time-points. Cells were washed with ice cold 1 × PBS, scraped and protein was isolated using NP-40 lysis buffer (50 mM Tris–HCL, 150 mM NaCl, 1% NP-40, 2 mM EDTA; Sigma-Aldrich, supplemented with 1 × PIC, 20 μM PR-619 DUBi and 1 × PhosSTOP) followed by sonication of 10 cycles of 30 s ON/30 s OFF using Bioruptor Pico sonication device. Lysates were clarified using 13,000 rpm centrifugation for 5 min and protein concentration was measured with PierceTM BCA Protein Assay Kit (Thermo Fisher Scientific, Waltham, MA, USA). 15 μg protein lysates were mixed with NuPAGE™ LDS Sample Buffer (4×) (Thermo Fisher Scientific, Waltham, MA, USA) and incubated at 95 °C for 10 min. Proteins were separated in precast Bolt™ 4–12% Bis–Tris Plus gradient gels (Thermo Fisher Scientific, Waltham, MA, USA). Proteins were transferred onto Amersham Hybond ECL-nitrocellulose membrane (GE Healthcare, Illinois, Chicago, USA) and blocked for 1 h at RT using 5% non-fat dry milk-TBST (Tris-Buffered Saline/0.1% Tween 20). Primary antibodies of CUL3 (ab194584; Abcam, Cambridge, UK), γH2AX (ab26350; Abcam, Cambridge, UK), α-Tubulin (T9026; Abcam, Cambridge, UK), pATM-S1981 (ab81292, Cambridge, UK), GFP (sc-8334; Santa Cruz Biotechnology, Dallas, Texas, USA), H3 (ab1791; Abcam, Cambridge, UK) and GAPDH (MAB374, Millipore, Burlington, MA, USA) were incubated overnight at 4 °C at 1:1000 dilution. Secondary antibodies of GAR-HRP IgG P0448 (Dako, Agilent Technologies, Santa Clara, CA, USA) or RAM-HRP IgG P0260 (Dako, Agilent Technologies, Santa Clara, CA, USA) were incubated with 1:5000 dilution and G:BOX Chemi XRQ (Syngene) system was used for chemiluminescent detection.

### Cell cycle analysis

3 × 10^5^ U2OS and 6 × 10^5^ MDA-MB-231 cells were seeded in a 6-well plate and 24 h later were transfected with 25 nM CUL3 siRNA SMARTpool (L-010224-00-0005; Dharmacon™ ON-Targetplus, Cambridge, UK) according to the manufacturers’ instructions. 48 h post transfection cells were treated with 50 ng/ml NCS for 6 h and collected with trypsinization in a microcentrifuge tube and fixed in 70% ethanol for 30 min on ice. Cells were pelleted and incubated with various concentrations of ethanol (50%, 30%, 10%) to remove ethanol slowly and were finally resuspended in 1 × PBS. Before FACS analysis, 1 μg/ml DAPI (Sigma-Aldrich, St. Louis, MO, USA) was added to the cell suspension and incubated for 30 min followed by cell analyses using BD FACS Aria Fusion flow cytometer.

### Quantitative real-time PCR

Total RNA was isolated by the ReliaPrep RNA Cell Miniprep System Kit (Promega, Madison, WI, USA) according to the manufacturer's instructions. RNA concentrations were determined by NanoDropTM OneC spectrophotometer (Thermo Fisher Scientific, Waltham, MA, USA) and reverse transcription was carried out using TaqMan® Reverse Transcription Reagents (Thermo Fisher Scientific, Waltham, MA, USA) according to the manufacturers’ instructions. Real-time quantitative PCR reactions using GoTaq® qPCR Master Mix (Promega, Madison, Wisconsin, USA) were performed with the QIAGEN Rotor-GeneQ 5-plex HRM qPCR System (Qiagen, Hilden, Germany) in a final volume of 10 µl using *CUL3* primers forward: 5′ CCTGCCCGCCTTAAATGTGACA 3′ and reverse: 5′ ATGGTCATCGGAAAGGCCC 3′. The following thermal profile condition was used for all RT-qPCR amplifications: 95 °C for 7 min, 45 cycles of 95 °C for 15 s and 60 °C for 30 s.

### GEPIA database analysis

Gene Expression Profiling Interactive Analysis (GEPIA) is a commonly used website used as a resource for gene expression profiles of given genes^40^. GEPIA, which contains 9736 tumor samples across 33 cancer types and 8587 normal tissues from the TCGA and the Genotype-Tissue Expression (GTEx) database, performs survival analysis based on gene expression levels according to user-defined sample selections and methods. We used GEPIA2 to find the expression level of the *CUL3* gene in distinct types of cancer with screening criteria q-value < 0.01 and |Log2FC| the cutoff point was 0.1 using ANOVA. For the overall survival (OS) analysis CUL3 was used as input filtering for breast cancer results in which the Hazard Ratio was calculated based on Cox PH model with 95% confidence interval and using median as group cutoff. The correlation between *CUL3* expression and survival, including (overall survival) OS in breast cancer was also analyzed by GEPIA.

### UALCAN database analysis

The University of Alabama at Birmingham Cancer data analysis Portal (UALCAN) is a comprehensive web resource for analyzing cancer OMICS data (TCGA, MET500, CPTAC and CBTTC). UALCAN provides access to graphs and plots depicting gene expression and survival curves, while recently it has provided access to data for microRNAs (miRNAs), long non-coding RNAs (lncRNAs), promoter DNA methylation from TCGA and mass spectrometry-based proteomics from the Clinical Proteomic tumor Analysis Consortium (CPTAC)^41,42^. In this study, we also used UALCAN to find patient survival information in breast cancer based on *CUL3* gene expression. To this end, CUL3 was used as a search input and breast cancer was selected to visualize the results. Since there was no option to customize thresholds and significance levels in the dataportal, all the parameters for the generation of the plots are being described in the publication of Chandrashekar et al., in 2017. We further used UALCAN to extract the plots for CUL3 expression across various tumor types as shown in Supplementary Fig. S1A top.

### Kaplan–Meier plotter

Kaplan–Meier plotter web resource was used to mine data for the overall survival of breast cancer patients with high/low expression of CUL3^43^. In Kaplan–Meier plotter gene expression data and relapse free and overall survival information are imported through GEO, EGA and TCGA. The database integrates gene expression and clinical data simultaneously. To analyze the prognostic value of a particular gene, the patient samples are split into two groups according to various quantile expressions of the proposed biomarker; in this case CUL3. To observe the survival data of CUL3 we mined into RNA-seq libraries filtering for CUL3 and breast cancer (1090 samples) from the Pancancer RNA-seq library. The two patient cohorts are compared by a Kaplan–Meier survival plot, with hazard ratio 1.48 with 95% confidence intervals and logrank *p* value 0.022. The patient groups were split according to the median value [low expression cohort with 148.53 median value (months) and high expression cohort with 108.73 median value (months)]. The Kaplan–Meier plot with CUL3 (Q13618) refers to protein expression data and overall survival with 65 total number of patients with hazard ratio 2.57 (1.23–5.35) with 95% confidence intervals and logrank *p* value 0.0089. The patient groups were split according to the median value [low expression cohort with 39 median value (months) and high expression cohort with 12 median value (months)].

### Functional enrichment analysis via EnrichR

Genes that were positively correlated with CUL3 with Pearson’s correlation coefficient ≥ 0.5 from UALCAN and GEPIA2 database as well as 12.454 disease target genes related to breast cancer according to GeneCards were used as input for DeepVenn and the intersection was submitted to EnrichR for gene ontology enrichment. EnrichR uniquely integrates knowledge from many high-profile projects to provide synthesized information about mammalian genes and gene sets^44–46^. The platform provides various methods to compute gene set enrichment and from the resulting gene set libraries we focused on the Gene Ontology Biological Process as well as Gene Ontology KEGG pathways. Top 10 pathways with the lowest *p* value scores (≤ 0.05) were chosen for further analysis.

### CUL3 expression correlation analysis across 27 tumor types

Genes positively correlating (Pearson’s correlation coefficient ≥ 0.3) with CUL3 in 27 tumor types were retrieved from UALCAN and together with the respective values of Pearson’s correlation coefficient per gene, they were used as input for Gene Set Enrichment Analysis (GSEA) which ranks the given genes based on a specified value; the correlation coefficient. For the Pearson's correlation coefficient a 0.3 cutoff value was applied to the gene lists to rule out very weakly correlating genes, yet still containing genes that are part of a network can have a biologically relevant effect^47^. GO gene set (c5.go.v2022.1.Hs.symbols.gmt) was used for background information from the Molecular Signatures Database (MSigDB)^48^. The pathway outputs from the GSEA with positive normalized enriched score (NES) and the most frequently enriched molecular signatures across all tumor types were selected and analyzed and plotted in heatmap using R studio while filtered for pathways with FDR q-value ≥ 0.1. The heatmap was generated with the ComplexHeatmap (version 2.18.0; https://bioconductor.org/packages/release/bioc/html/ComplexHeatmap.html) R (R version 4.3.0) package using GO terms that were enriched in at least 10 different tumor types^49,50^. Hierarchical clustering of rows and columns was based on Euclidean distance.

### In house TCGA data analysis

Breast cancer (BRCA) and Diffuse large B cell lymphoma (DLBC) primary tumor expression (TPM, raw STAR counts) and clinical data was downloaded from The Cancer Genome Atlas (TCGA) using the TCGAbiolinks R package (TCGAbiolinks version 2.30.0)^51–53^. The following parameters were used within the GDCquery() command; project = TCGA-BRCA and TCGA-DLBC respectively, data.category = Transcriptome Profiling, sample.type = Primary tumor, data.type = Gene Expression Quantification, access = open, workflow.type = STAR-Counts. This data was then downloaded using GDCdownload(), and stored in an R object with data = GDCprepare(query = query, summarizedExperiment = T). TPM values were retrieved using data@assays@data@listData$tpm_unstranded. Raw STAR counts were retrieved by selecting columns containing the unstranded raw count values. Samples with missing PAM50 or stage classification were omitted from further analysis. Stage information was merged into four groups to account for the low number of samples in some of the categories: stage I (Stage I, stage Ia, stage Ib), stage II (stage II, stage IIa, stage IIb), stage III (Stage III, stage IIIa, stage IIIb, stage IIIc), stage IV.

Normal (breast-mammary and spleen) tissue TPM values and raw count were downloaded directly from The Genotype-Tissue Expression (GTEx) project portal: https://www.gtexportal.org/home/downloads/adult-gtex. (gene_reads_2017-06-05_v8_breast_mammary_tissue.gct.gz, gene_reads_2017-06-05_v8_spleen.gct.gz)^54^.

Significance between mean TPM values were calculated by Student's t-Test in R (*p* < 0.1*, *p* < 0.01**, *p* < 0.001***, *p* < 0.0001****, *p* > 0.1 ns).

CUL3 over- and under expression was determined based on TPM values. Cutoff values were chosen so that there’s a minimal overlap in the CUL3 TPM values between CUL3 over or under expressing and normal samples, moreover sample sizes in the over and under expressing groups are comparable (Table 1).

**Table 1:** TCGA-BRCA cutoff values without filtering for PAM50 subtype.

CUL3 under expressing samples	TPM < 11
CUL3 over expressing samples	TPM > 37
**TCGA-BRCA LumA cutoff values**	
CUL3 under expressing samples	TPM < 19
CUL3 over expressing samples	TPM > 31
**TCGA-BRCA Basal cutoff values**	
CUL3 under expressing samples	TPM < 12
CUL3 over expressing samples	TPM > 22
**TCGA-DLBC cutoff values**	
CUL3 under expressing samples	TPM < 11
CUL3 over expressing samples	TPM > 17

Differential gene expression (DEG) analyses were conducted with the DESeq2 (version 1.38.3) R package setting *p* value threshold to 0.01^55^. DESeqDataSet objects were created for the CUL3 over expressing, LumA CUL3 over expressing and Basal CUL3 over expressing categories using the raw count values corresponding to these samples. These sample groups were all contrasted against the GTEx mammary tissue raw counts as was previously performed in Huey-Minn et al.^56^. The DESeq() function was used with adjusted *p* value (< 0.01) and log2 fold change (> 1 and < − 1) cutoff values to obtain significantly differentially expressed gene lists. These filtered gene lists ordered by the log2 fold changes served as input to the gene-set enrichment analyses. Gene set enrichment analysis (GSEA) was conducted with the R package fgsea (version 1.28.0) (minSize = 5, maxSize = 500)^57^. Hallmark and Gene Ontology gene sets were used for background information from the MSigDB (h.all.v2022.1.Hs.symbols.gmt, c5.go.v2022.1.Hs.symbols.gmt)^48^.

Survival data was retrieved from TCGA and analyzed with the help of RTCGA (version 1.32.0), RTCGA.clinical (version 20151101.32.), survminer (version 0.4.9) and survival (version 3.5-5) R packages^58–61^. Significance levels were evaluated based on the Cox Proportional Hazards Regression Analysis using the coxph() function from the survival package^62^. The survival plots were created with the ggsurvfit (version 1.0.0) R package^63^.

### Supplementary Information

Supplementary Information 1.

Supplementary Video 1.

## Data Availability

Breast cancer (BRCA) and Diffuse large B cell lymphoma (DLBC) primary tumor expression (TPM, raw STAR counts) and clinical data was downloaded from The Cancer Genome Atlas (TCGA) using the TCGAbiolinks R package and Normal (breast-mammary and spleen) tissue TPM values were downloaded directly from The Genotype-Tissue Expression (GTEx) project portal from the following links: https://gtexportal.org/home/downloads/adult-gtex/bulk_tissue_expression File names: (gene_reads_2017-06-05_v8_breast_mammary_tissue.gct.gz, gene_reads_2017-06-05_v8_spleen.gct.gz).

## References

[1] Breast Cancer. *Breast Cancer Information and Overview*. American Cancer Society. https://www.cancer.org/cancer/types/breast-cancer.html.

[2] Roy M, Fowler AM, Ulaner GA, Mahajan A (2020). Molecular classification of breast cancer. PET Clin..

[3] Pankotai-Bodó G, Oláh-Németh O, Sükösd F, Pankotai T (2023). Routine molecular applications and recent advances in breast cancer diagnostics. J. Biotechnol..

[4] Timbres J (2023). DCIS and LCIS: Are the risk factors for developing in situ breast cancer different?. Cancers (Basel).

[5] Types of Breast Cancer. BCRF. https://www.bcrf.org/blog/types-of-breast-cancer/.

[6] Shien T, Iwata H (2020). Adjuvant and neoadjuvant therapy for breast cancer. Jpn. J. Clin. Oncol..

[7] Rossi L, Mazzara C, Pagani O (2019). Diagnosis and treatment of breast cancer in young women. Curr. Treat. Options Oncol..

[8] Hollingsworth AB (2019). Redefining the sensitivity of screening mammography: A review. Am. J. Surg..

[9] Lee Y (2023). Recent advances of small extracellular vesicle biomarkers in breast cancer diagnosis and prognosis. Mol. Cancer.

[10] Borsos BN (2022). BC-miR: monitoring breast cancer-related MiRNA profile in blood sera—A prosperous approach for tumor detection. Cells.

[11] Han D, Wang L, Jiang S, Yang Q (2023). The ubiquitin–proteasome system in breast cancer. Trends Mol. Med..

[12] Li S, Zhang H, Wei X (2021). Roles and mechanisms of deubiquitinases (DUBs) in breast cancer progression and targeted drug discovery. Life (Basel, Switzerland).

[13] Carlucci A, D’Angiolella V (2015). It is not all about BRCA: Cullin-ring ubiquitin ligases in ovarian cancer. Br. J. Cancer.

[14] Ioris RM, Ferris K, D’Angiolella V, Cromm P (2023). E3 ubiquitin ligases as molecular machines and platforms for drug development. Inducing Targeted Protein Degradation.

[15] Borsos BN, Majoros H, Pankotai T (2020). Ubiquitylation-mediated fine-tuning of DNA double-strand break repair. Cancers (Basel).

[16] Popovic D, Vucic D, Dikic I (2014). Ubiquitination in disease pathogenesis and treatment. Nat. Med..

[17] Harper JW, Schulman BA (2021). Cullin-RING ubiquitin ligase regulatory circuits: A quarter century beyond the F-box hypothesis. Annu. Rev. Biochem..

[18] Haagenson KK (2012). Cullin-3 protein expression levels correlate with breast cancer progression. Cancer Biol. Ther..

[19] Zhou Z (2021). BCAS3 exhibits oncogenic properties by promoting CRL4A-mediated ubiquitination of p53 in breast cancer. Cell Prolif..

[20] Deng J (2022). CRL4-DCAF8L2 E3 ligase promotes ubiquitination and degradation of BARD1. Biochem. Biophys. Res. Commun..

[21] Murakami A (2019). Cullin-3/KCTD10 E3 complex is essential for Rac1 activation through RhoB degradation in human epidermal growth factor receptor 2-positive breast cancer cells. Cancer Sci..

[22] Liu A, Zhang S, Shen Y, Lei R, Wang Y (2019). Association of mRNA expression levels of Cullin family members with prognosis in breast cancer: An online database analysis. Medicine (United states).

[23] Soule HD, Vazquez J, Long A, Albert S, Brennan M (1973). A human cell line from a pleural effusion derived from a breast carcinoma. JNCI J. Natl. Cancer Inst..

[24] Lee AV, Oesterreich S, Davidson NE (2015). MCF-7 cells–changing the course of breast cancer research and care for 45 years. J. Natl. Cancer Inst..

[25] So JY, Ohm J, Lipkowitz S, Yang L (2022). Triple negative breast cancer (TNBC): Non-genetic tumor heterogeneity and immune microenvironment: Emerging treatment options. Pharmacol. Ther..

[26] Fedele M, Sgarra R, Battista S, Cerchia L, Manfioletti G (2022). The epithelial–mesenchymal transition at the crossroads between metabolism and tumor progression. Int. J. Mol. Sci..

[27] Wang R, Nakshatri H (2020). Systemic actions of breast cancer facilitate functional limitations. Cancers (Basel).

[28] Jagannathan V, Robinson-Rechavi M (2011). Meta-analysis of estrogen response in MCF-7 distinguishes early target genes involved in signaling and cell proliferation from later target genes involved in cell cycle and DNA repair. BMC Syst. Biol..

[29] Chen HY, Chen RH (2016). Cullin 3 ubiquitin ligases in cancer biology: Functions and therapeutic implications. Front. Oncol..

[30] Li X (2021). CUL3 (cullin 3)-mediated ubiquitination and degradation of BECN1 (beclin 1) inhibit autophagy and promote tumor progression. Autophagy.

[31] Loignon M (2009). Cul3 overexpression depletes Nrf2 in breast cancer and is associated with sensitivity to carcinogens, to oxidative stress, and to chemotherapy. Mol. Cancer Ther..

[32] Kim K, Kim YJ (2022). RhoBTB3 regulates proliferation and invasion of breast cancer cells via Col1a1. Mol. Cells.

[33] Choi YM (2016). DBC2/RhoBTB2 functions as a tumor suppressor protein via Musashi-2 ubiquitination in breast cancer. Oncogene.

[34] Cummings CM, Bentley CA, Perdue SA, Baas PW, Singer JD (2009). The Cul3/Klhdc5 E3 ligase regulates p60/Katanin and is required for normal mitosis in mammalian cells. J. Biol. Chem..

[35] Jang SM, Redon CE, Thakur BL, Bahta MK, Aladjem MI (2020). Regulation of cell cycle drivers by Cullin-RING ubiquitin ligases. Exp. Mol. Med..

[36] McEvoy JD, Kossatz U, Malek N, Singer JD (2007). Constitutive turnover of cyclin E by Cul3 maintains quiescence. Mol. Cell. Biol..

[37] Tao S, Rojo de la Vega M, Chapman E, Ooi A, Zhang DD (2018). The effects of NRF2 modulation on the initiation and progression of chemically and genetically induced lung cancer. Mol. Carcinog..

[38] Chen RH (2020). Cullin 3 and Its role in tumorigenesis. Adv. Exp. Med. Biol..

[39] Kitamura H, Motohashi H (2018). NRF2 addiction in cancer cells. Cancer Sci..

[40] Tang Z (2017). GEPIA: a web server for cancer and normal gene expression profiling and interactive analyses. Nucleic Acids Res..

[41] ShimogaChandrashekar D (2022). UALCAN: An update to the integrated cancer data analysis platform. Neoplasia.

[42] Chandrashekar DS (2017). UALCAN: A portal for facilitating tumor subgroup gene expression and survival analyses 1. Neoplasia.

[43] Győrffy B (2023). Discovery and ranking of the most robust prognostic biomarkers in serous ovarian cancer. GeroScience.

[44] Chen EY (2013). Enrichr: Interactive and collaborative HTML5 gene list enrichment analysis tool. BMC Bioinform..

[45] Kuleshov MV (2016). Enrichr: a comprehensive gene set enrichment analysis web server 2016 update. Nucleic Acids Res..

[46] Xie Z (2021). Gene set knowledge discovery with enrichr. Curr. Protoc..

[47] Ratner B (2009). The correlation coefficient: Its values range between 1/1, or do they. J. Target. Meas. Anal. Mark..

[48] Liberzon A (2015). The molecular signatures database (MSigDB) hallmark gene set collection. Cell Syst..

[49] Gu Z, Eils R, Schlesner M (2016). Complex heatmaps reveal patterns and correlations in multidimensional genomic data. Bioinformatics.

[50] Gu Z (2022). Complex heatmap visualization. iMeta.

[51] Silva TC (2016). TCGA workflow: Analyze cancer genomics and epigenomics data using Bioconductor packages. F1000Research.

[52] Colaprico A (2016). TCGAbiolinks: an R/bioconductor package for integrative analysis of TCGA data. Nucleic Acids Res..

[53] Mounir M (2019). New functionalities in the TCGAbiolinks package for the study and integration of cancer data from GDC and GTEx. PLoS Comput. Biol..

[54] Lonsdale J (2013). The genotype-tissue expression (GTEx) project. Nat. Genet..

[55] Love MI, Huber W, Anders S (2014). Moderated estimation of fold change and dispersion for RNA-seq data with DESeq2. Genome Biol..

[56] Chen HM, MacDonald JA (2022). Network analysis of TCGA and GTEx gene expression datasets for identification of trait-associated biomarkers in human cancer. STAR Protoc..

[57] Korotkevich G (2021). Fast gene set enrichment analysis. bioRxiv.

[58] Kosinski, M. M. *Package ‘RTCGA’ Title The Cancer Genome Atlas Data Integration* (2023).

[59] Kassambara, A., Kosinski, M. & Biecek, P. *Drawing Survival Curves using ‘ggplot2’ [R package survminer version 0.4.8]* (2020).

[60] Therneau, T. *A Package for Survival Analysis in R* (2023).

[61] Kosinski, M. M. *Type Package Title Clinical datasets from The Cancer Genome Atlas Project* (2016).

[62] Therneau TM, Grambsch PM (2000). Modeling Survival Data: Extending the Cox Model.

[63] ggsurvfit: Flexible Time-to-Event Figures version 1.0.0 from CRAN. https://rdrr.io/cran/ggsurvfit/.

