# Supplementary material for: Prognostic potential of CUL3 ligase with differential roles in luminal A and basal type breast cancer tumors
Source: Sci Rep. 2024 Jun 28;14:14912. doi: 10.1038/s41598-024-65692-z (PMC11213933; doi:10.1038/s41598-024-65692-z)
Supplement: Supplementary file 1 — Supplementary Information 1. [file 41598_2024_65692_MOESM1_ESM.docx]

**Supplementary information**


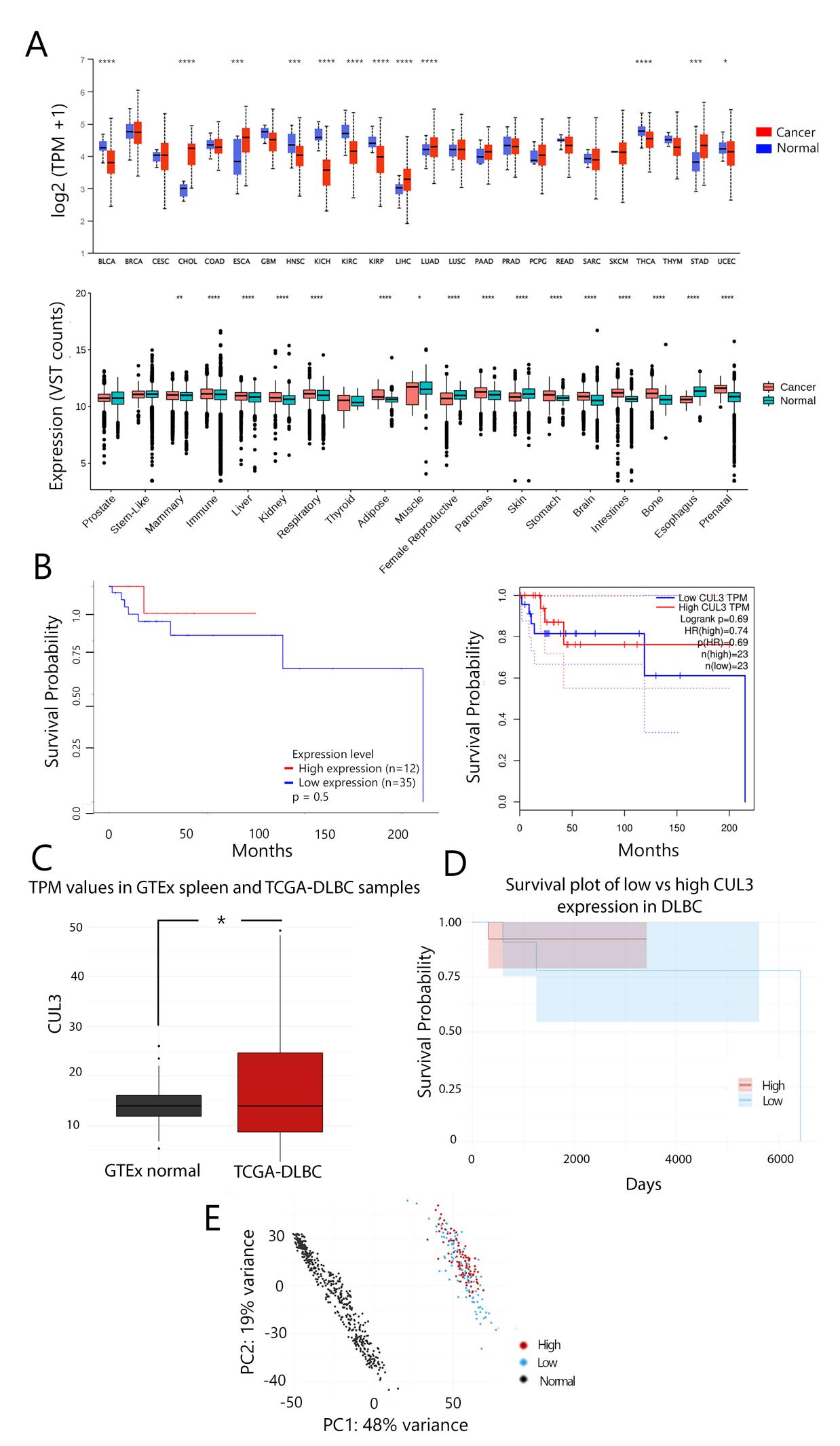


Supplementary Fig S1. A (*up*) Boxplot of CUL3 expression [Transcript per million values (TPM)] in 24 different tumor and normal samples  retrieved from UALCAN webportal and (*down*) Boxplot of CUL3 expression [Variance stabilising transformation (VST counts)] in 19 tumor and normal tissues retrieved from GEPIA2 webportal, B Kaplan-meier plot of high versus low CUL3 expression of DLBC patients calculated and retrieved from UALCAN (*left*) and Kaplan-meier plotter data source (*right*), C Boxplot of “in-house TCGA analysis” of CUL3 expression in normal versus DLBC tumor samples, D Survival kaplan-meier plot of low versus high CUL3 expression of “in-house TCGA analysis”, and E Principal component analysis (PCA) of normal, low and high CUL3 BRCA samples. The PCA values were calculated based on the VST transformed read counts from the raw data. Asterisks from the A *top* were calculated and retrieved as values from UALCAN while the authors denoted the asterisks as follows: *0.0332, **0.0021, ***0.0002, ****<0.0001. Asterisks from the A *down* were calculated and retrieved directly from GEPIA2.


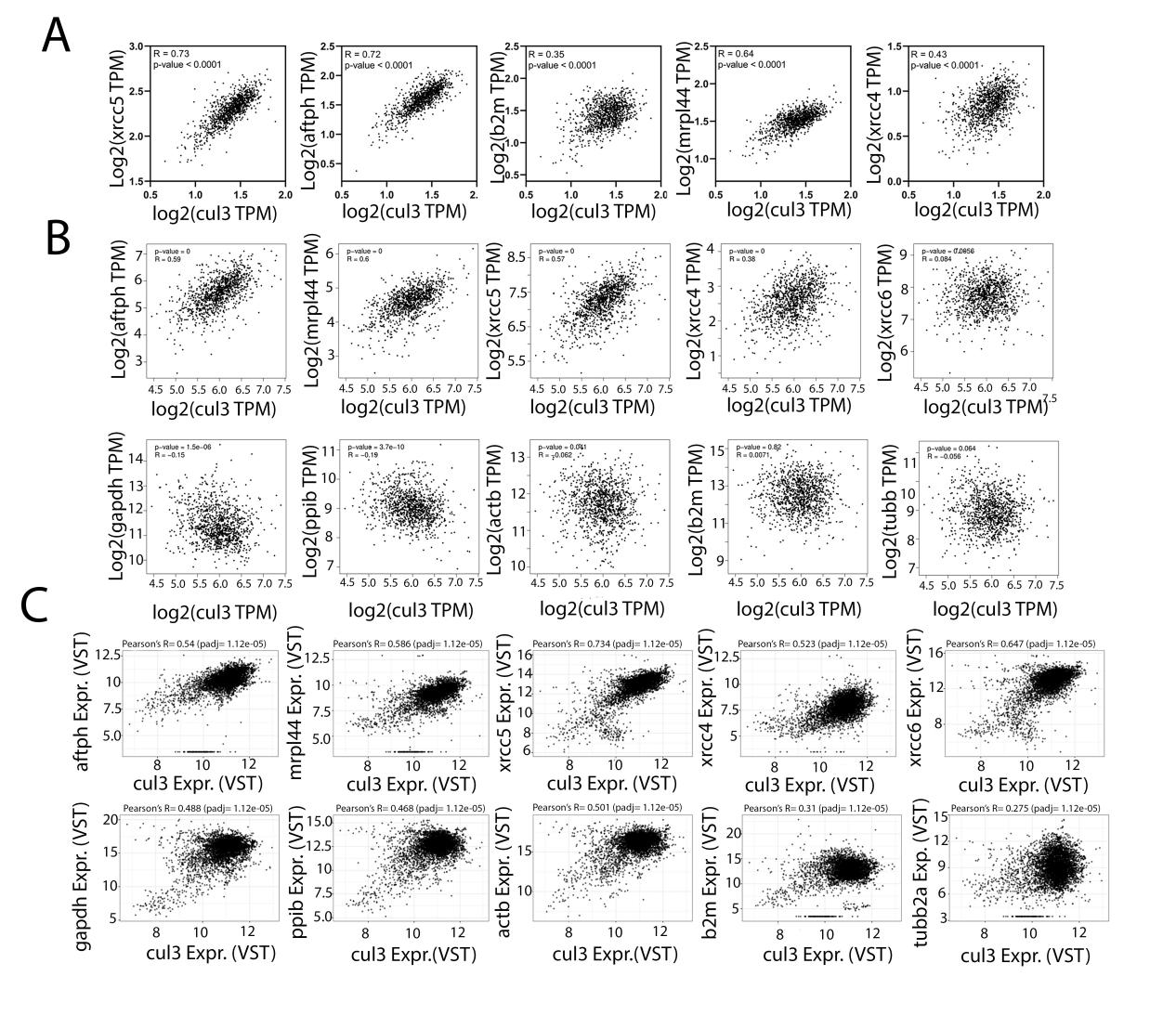


Supplementary Fig S2.  Co-expression correlation analysis of *CUL3* and different genes depicted in the graphs (*XRCC5, AFTPH, MRPL44, XRCC4, XRCC6, GAPDH, B2M, PPIB, ACTB, TUBB2A*), plots retrieved from (A) UALCAN, (B) GEPIA2 and (C) AnalyzeR. Pearson’s correlations coefficient R, and p-values are depicted on each graph and were calculated from the respective data portals.


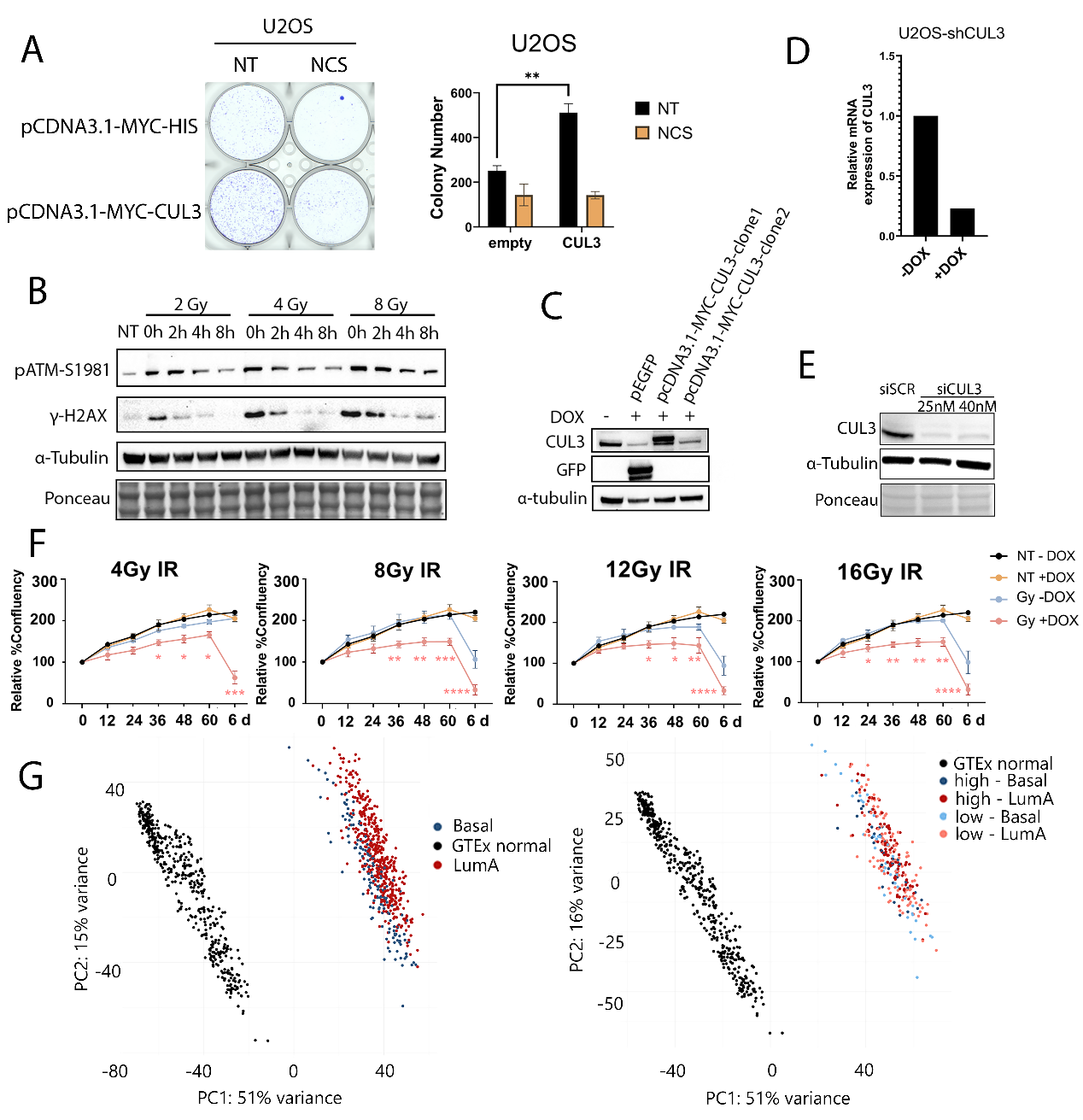


Supplementary Fig S3. A *left* Colony formation assay on U2OS cell line after overexpression of MYC-HIS (empty) vector and MYC-CUL3 as well as treatment with 100ng/ml NCS and *Right* quantification of colonies and statistical analysis using Tukey’s two-way Anova test of three biological replicates, B Immunoblotting analysis showing the induction of  two markers of DSB induction (pATM-S1981 and γH2AX) after 2, 4 and 8 Gy of IR at the indicated time points, C Immunoblotting analysis after DOX treatment in U2OS-shCUL3 cell line and overexpression of CUL3, D Relative mRNA expression of CUL3 after DOX-induced knock-down, E Immunoblotting analysis of siRNA transient transfection against CUL3 using 25nM and 40nM concentration, and F Cell proliferation assay after DOX-induced shCUL3 knock-down and increasing IR dosages in U2OS-shCUL3 cell line and G Principal component analysis (PCA) of (*left*) normal, Luminal A and Basal BRCA types and (*right*) normal, low and high CUL3 Luminal A and low and high Basal BRCA samples. The PCA values were calculated based on the VST transformed read counts from the raw data. All quantifications were performed using GraphPad Prism version 8.4.3 software (https://www.graphpad.com/) and Tukey’s test two-way Anova was applied for statistical analysis for which p-value was considered significant as follows:  *0.0332, **0.0021, ***0.0002, ****<0.0001

**
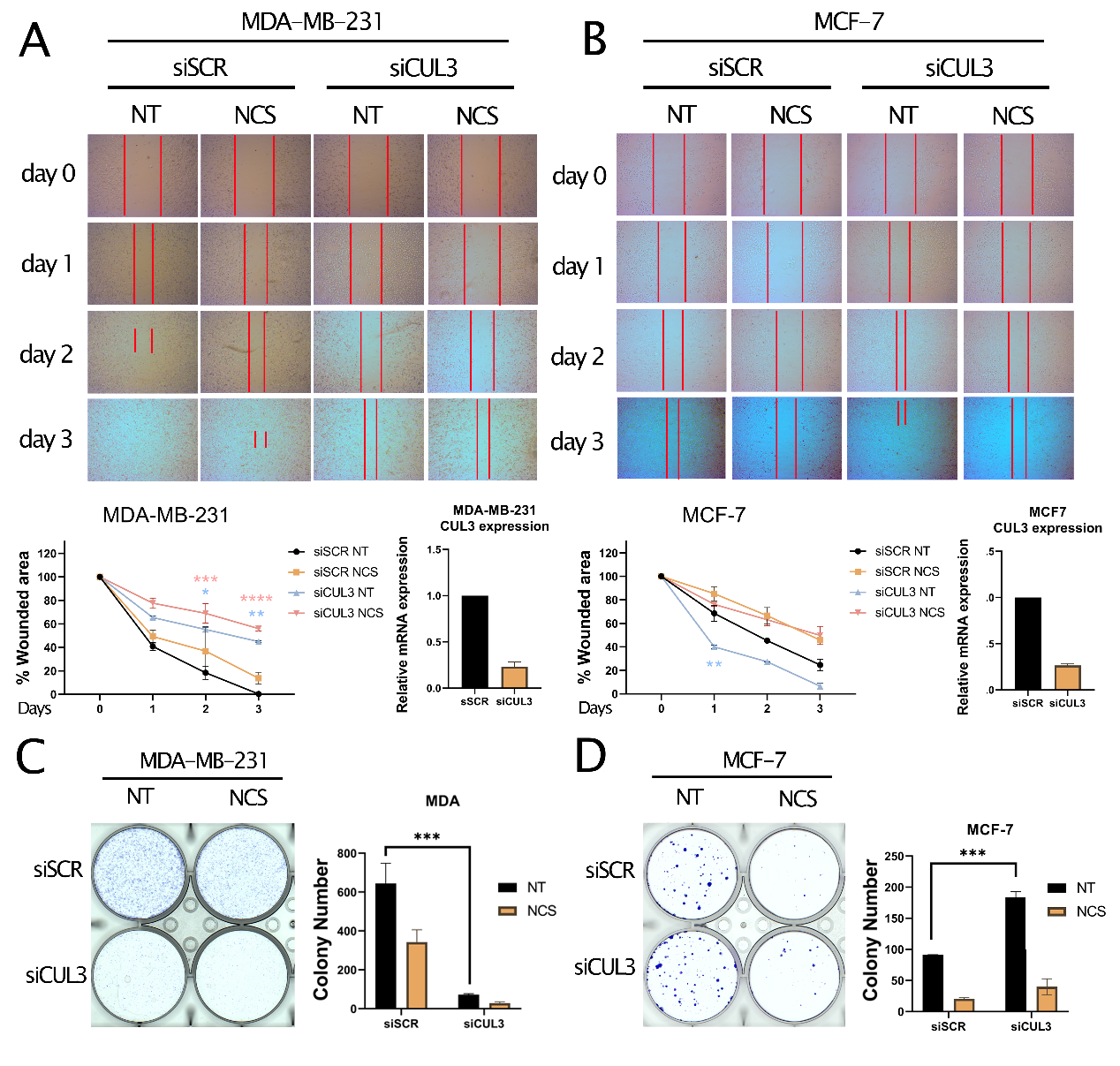
**

Supplementary Fig S4. A Microscopic images of wound healing assay in MDA-MB-231 cells and B in MCF-7 cell line after siCUL3 knock-down and neocarzinostatin (NCS) treatment and bottom quantification of the wounded area and CUL3 silencing efficiency measured by qPCR, C colony formation assay of MDA-MB-231 cells and D in MCF-7 cells and quantification on the right

**
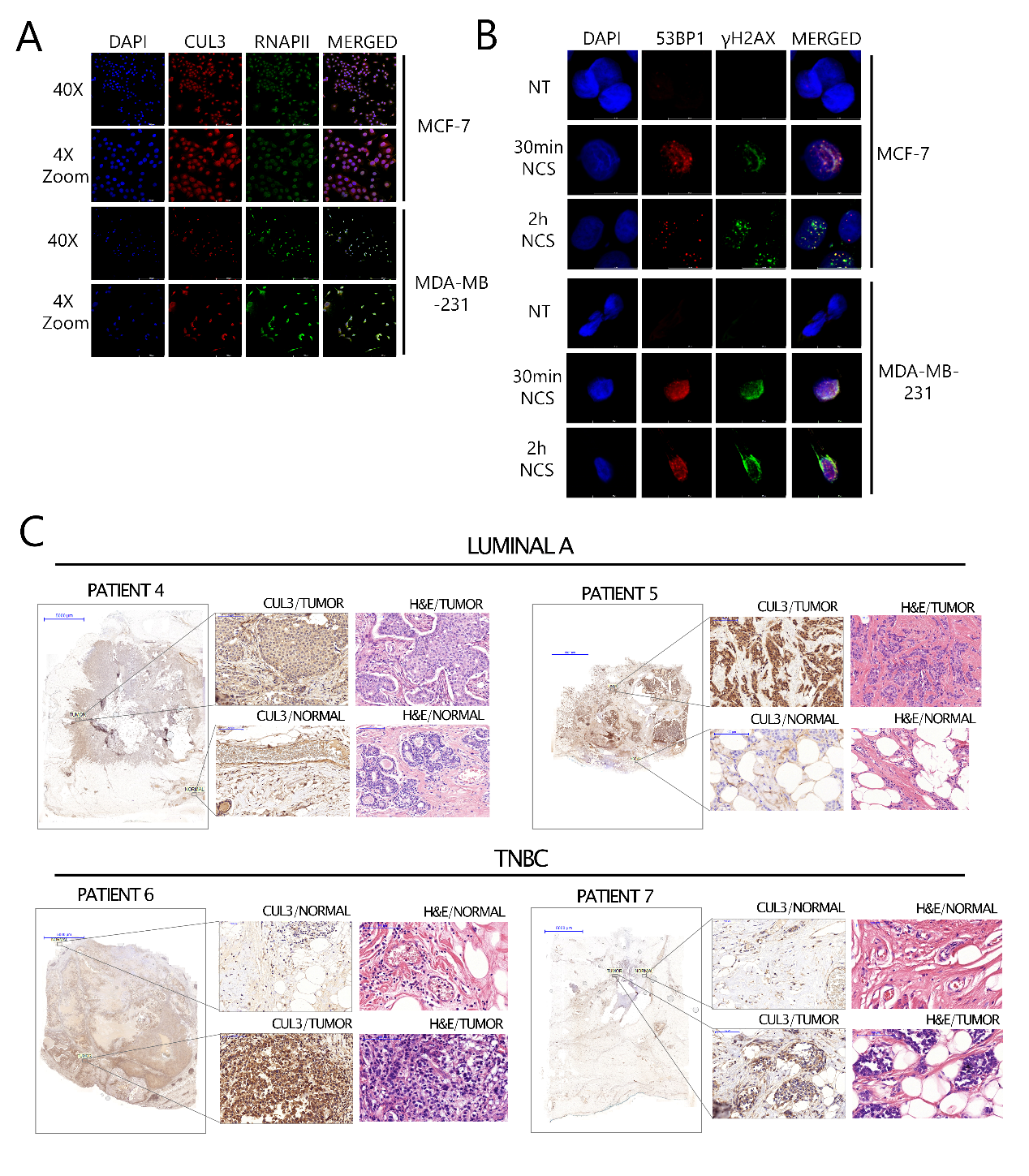
**

Supplementary Fig S5. A Representative images of CUL3 and RNAPII localization in MDA-MB-231 and MCF-7 cells after immunostaining, B Representative images of γH2AX and 53BP1 co-localization after 30min or 2h post neocarzinostatin (NCS) treatment after immunostaining MDA-MB-231 and MCF-7 cells (DAPI counterstaining nuclei) and C CUL3 expression and localization localization in breast tissues of luminal A (top) and TNBC (bottom) after immunohistochemistry (IHC) with DAB chromogen staining (brown; staining CUL3) and Hematoxylin (blue; counter staining nuclei) with parallel Hematoxylin and Eosin (H&E) staining of the same tissue


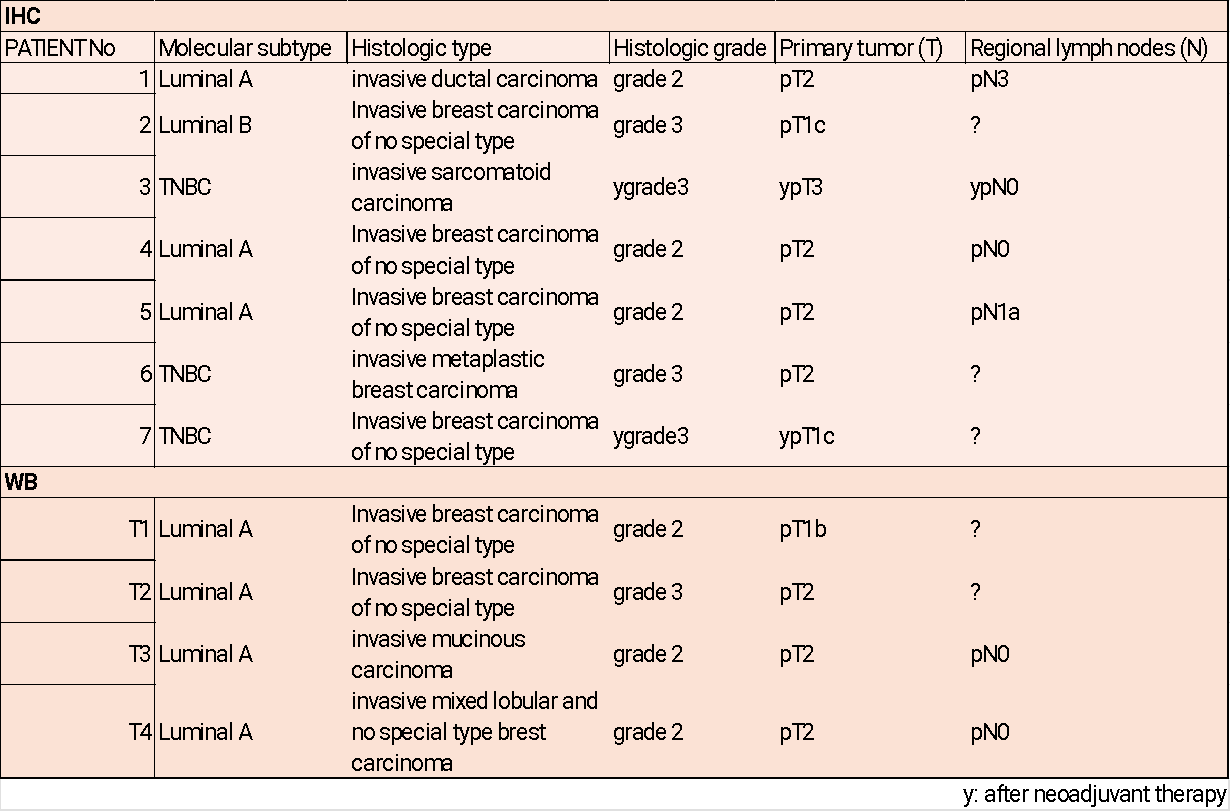


Supplementary Table S1. Patient data used in this study. Molecular subtype, Histologic type and grade, Primary Tumor (T) and Regional lymph nodes (N) location are depicted on the table above.

**Supplementary Video 1.**

Monitoring survival of U2OS- shCUL3 cell line in 6 days time-course using IncuCyte S3 Live-Cell Analysis System. Both panels show cells treated with 1μg/ml DOX, left panel shows cells with no irradiation and right panel cells with 8Gy IR.
